# Supplementary material for: An in situ dual-anchoring strategy for enhanced immobilization of PD-L1 to treat autoimmune diseases
Source: Nat Commun. 2023 Oct 31;14:6953. doi: 10.1038/s41467-023-42725-1 (PMC10618264; doi:10.1038/s41467-023-42725-1)
Supplement: Supplementary file 1 — Supplementary Information [file 41467_2023_42725_MOESM1_ESM.pdf]

Supplementary Materials

An *In Situ* Dual-Anchoring Strategy for Enhanced Immobilization of PD-L1 to Treat Autoimmune Diseases

Shenqiang Wang[1], Ying Zhang[1], Yanfang Wang[1], Yinxian Yang[1], Sheng Zhao[1], Tao Sheng[1], Yuqi Zhang[1,2,3], Zhen Gu[1,3,4,5,6,7]✉, Jinqiang Wang[1,3,6,8]✉, Jicheng Yu[1,3,4,5,6,]✉

[1]Zhejiang Provincial Key Laboratory for Advanced Drug Delivery Systems, College of Pharmaceutical Sciences, Zhejiang University, Hangzhou 310058, China.

[2]Department of Burns and Wound Center, Second Affiliated Hospital, School of Medicine, Zhejiang University, Hangzhou 310009, China.

[3]National Key Laboratory of Advanced Drug Delivery and Release Systems, Zhejiang University, Hangzhou 310058, China.

[4]Liangzhu Laboratory, Zhejiang University Medical Center, Hangzhou 311121, China.

[5]Department of General Surgery, Sir Run Run Shaw Hospital, School of Medicine, Zhejiang University, Hangzhou 310016, China.

[6]Jinhua Institute of Zhejiang University, Jinhua 321299, China.[7]MOE Key Laboratory of Macromolecular Synthesis and Functionalization, Department of Polymer Science and Engineering, Zhejiang University, Hangzhou 310027, China.

[8]Department of Pharmacy, Second Affiliated Hospital, Zhejiang University School of Medicine, Zhejiang University, Hangzhou 310009, China.

✉Corresponding authors. Email: guzhen@zju.edu.cn; jinqiang_wang@zju.edu.cn; yujicheng@zju.edu.cn

Supplementary Figures

Supplementary Figure 1. Synthetic routes of $MPEG_{5k}$-$P(DMAEMA)_{6k}$.

Supplementary Figure 2. Synthetic route and ^{1}H-NMR spectra of $MPEG_{5k}$-$P(DMAEMA$-$PBA)_{14k}$.

Supplementary Figure 3. The drug loading capacity of NHS-PEG-PLGA nanoparticles, MPEG-P(DMAEMA-PBA) nanoparticles, and H-NPs.

Supplementary Figure 4. ROS-responsive degradation behavior of H-NPs.

Supplementary Figure 5. The cytotoxicity of $Ac_4ManNAz$ and H-NPs with different concentrations.

Supplementary Figure 6. The characterization of PD-L1 analogs.

Supplementary Figure 7. The immobilization performance of PD-L1 analogs on the cell membrane.

Supplementary Figure 8. The dual-anchor coupling strategy prolonged the immobilization of PD-L1 on the cell membrane.

Supplementary Figure 9. The dual-anchor coupling strategy enhanced the expression of PD-L1 on Min 6 cells.

Supplementary Figure 10. Gating strategy for T cells analysis by flow cytometry in the *in vitro* experiment.

Supplementary Figure 11. Characterizations of the status of anti-PD-1-treated T cells in different treatment groups analyzed by flow cytometry.

Supplementary Figure 12. Treg cell suppressive activity.

Supplementary Figure 13. GLP1R-H-NPs could be preferably internalized into pancreatic cells.

Supplementary Figure 14. GLP1R facilitated H-NPs delivery to the pancreas of NOD mice.

Supplementary Figure 15. Histological analysis of liver.

Supplementary Figure 16. The dual-anchor coupling strategy prolonged the immobilization of PD-L1 on islets.

Supplementary Figure 17. Body weights of the diabetic NOD mice with different treatments.

Supplementary Figure 18. PD-L1 bioengineering reverses the early-onset type 1 diabetes in the NOD mice.

Supplementary Figure 19. Representative plots and quantification of pancreas-infiltrating $CD3^+$ T cells in different treatment groups analyzed by flow cytometry.

Supplementary Figure 20. Characterization of $CD4^+$ T cells in the pancreas of NOD mice.

Supplementary Figure 21. Quantification of pancreas-infiltrating CD4$^+$ T cells.
Supplementary Figure 22. Characterizations of the T-cell status in the pancreas of diabetic NOD mice.
Supplementary Figure 23. Characterization of Treg cells in the pancreas of NOD mice.
Supplementary Figure 24. Representative plots and quantification of pancreas-infiltrating FoxP3$^+$ T cells in different treatment groups analyzed by flow cytometry.
Supplementary Figure 25. Representative plots and quantification of pancreas-infiltrating CD4$^+$CD49b$^+$ T cells in different treatment groups analyzed by flow cytometry.
Supplementary Figure 26. Gating strategy for T cells analysis by flow cytometry in the lymph node (LN) of NOD mice.
Supplementary Figure 27. Characterizations of the T-cell status in the lymph node (LN) of diabetic NOD mice.
Supplementary Figure 28. Gating strategy for T cells analysis by flow cytometry in the spleen of NOD mice.
Supplementary Figure 29. Characterizations of the T-cell status in the spleen of diabetic NOD mice.
Supplementary Figure 30. Confocal imaging of chondrocytes labeled with FITC-labeled PD-L1 analogs on day 3.
Supplementary Figure 31. Representative 3D-reconstructed micro-CT images of left hind ankle joints of normal and arthritic mice after different treatments on day 48.
Supplementary Figure 32. *In situ* immobilization of PD-L1 ameliorates left joint swelling.
Supplementary Figure 33. The average Clinical score of arthritis in mice with different treatments.
Supplementary Figure 34. Representative anti-CD4, anti-CD8, and anti-FoxP3 (red) stained knee sections from DBA mice after different treatments.
Supplementary Figure 35. The T cell status in the spleen of DBA mice.
Supplementary Figure 36. Representative anti-CD206 (green) and anti-CD86 (red) stained knee sections from DBA mice after different treatments.
Supplementary Figure 37. *In situ* immobilization of PD-L1 ameliorates joint disruption.
Supplementary Figure 38. *In situ* immobilization of PD-L1 ameliorates digital joint disruption.
Supplementary Figure 39. Several key hematological, hepatic, and renal parameters of DBA mice with different treatments.
Supplementary Figure 40. *In vivo* cytotoxicity evaluation.

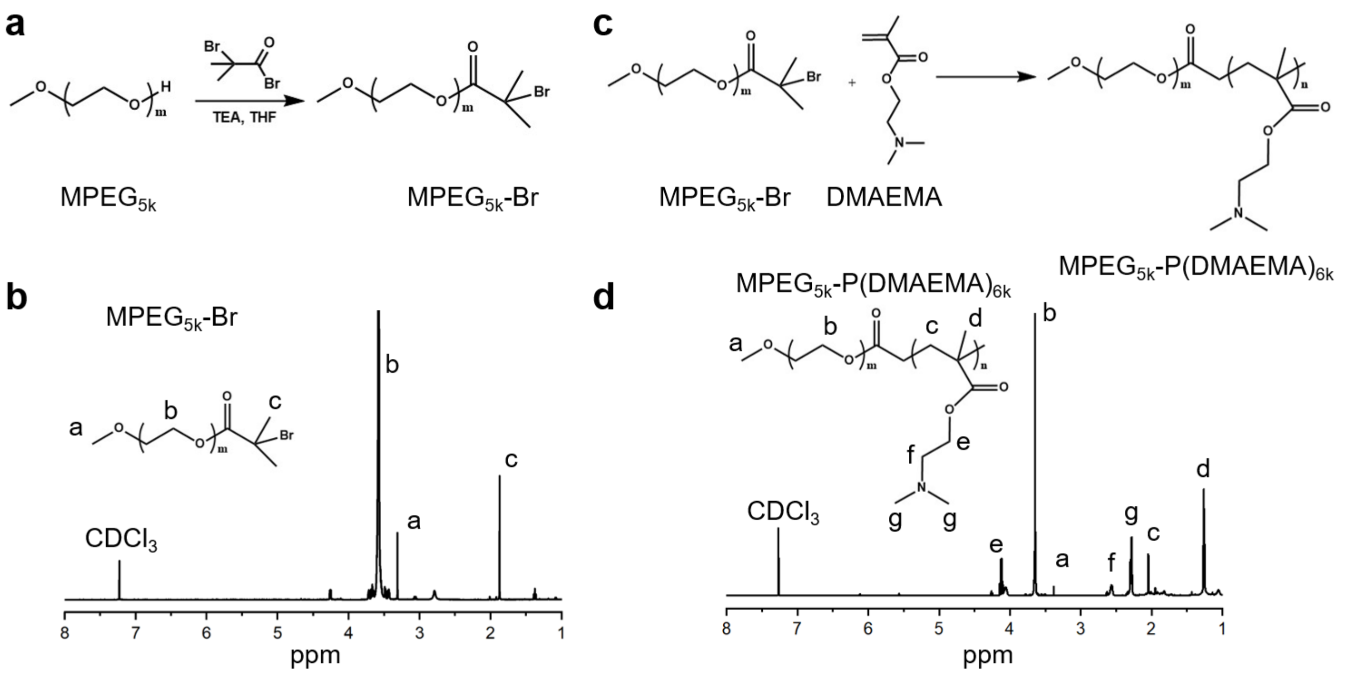

Supplementary Figure 1. Synthetic routes of MPEG$_{5k}$-P(DMAEMA)$_{6k}$. a,**b**, Synthetic route (**a**) and ^{1}H-NMR spectra (**b**) of MPEG$_{5k}$-Br. **c**,**d**, Synthetic route (**c**) and ^{1}H-NMR spectra (**d**) of MPEG$_{5k}$-P(DMAEMA)$_{6k}$. ^{1}H NMR (500 MHz, $CDCl_3$) of MPEG$_{5k}$-Br: δ 3.58 (s, 226H), 3.31 (s, 3H), 1.90 (d, J = 23.0 Hz, 6H). ^{1}H NMR (500 MHz, $CDCl_3$) of MPEG$_{5k}$-P(DMAEMA)$_{6k}$: δ 4.30-3.95 (m, 76H), 3.64 (s, 226H), 3.38 (s, 3H), 2.64-2.50 (m, 76H), 2.36-2.21 (m, 228H), 2.05 (s, 76H), 1.26 (dd, J = 8.6, 5.7 Hz, 114H).

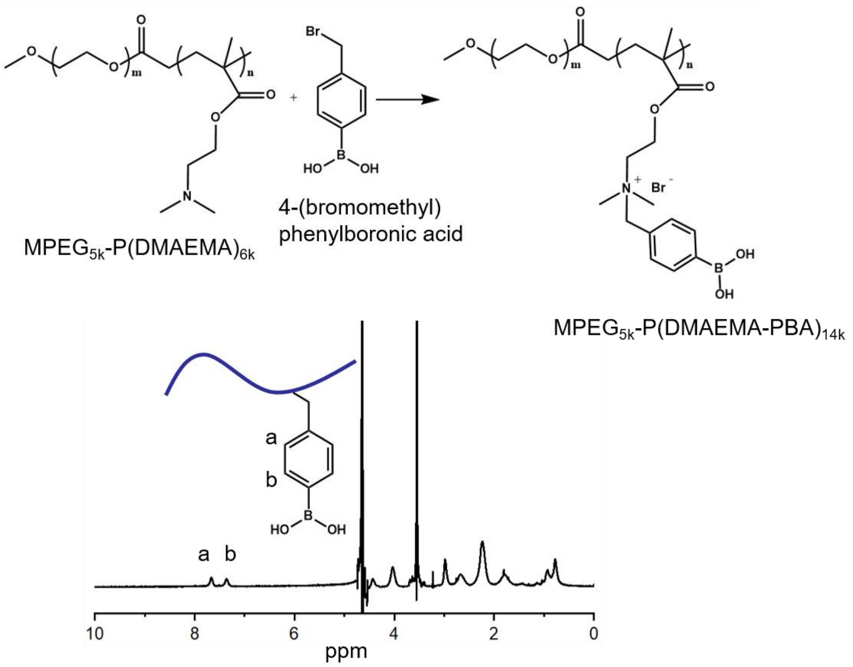

Supplementary Figure 2. Synthetic route and ^{1}H-NMR spectra of MPEG$_{5k}$-P(DMAEMA-PBA)$_{14k}$. ^{1}H NMR (500 MHz, D_2O) δ 7.72 (s, 2H), 7.43 (s, 2H).

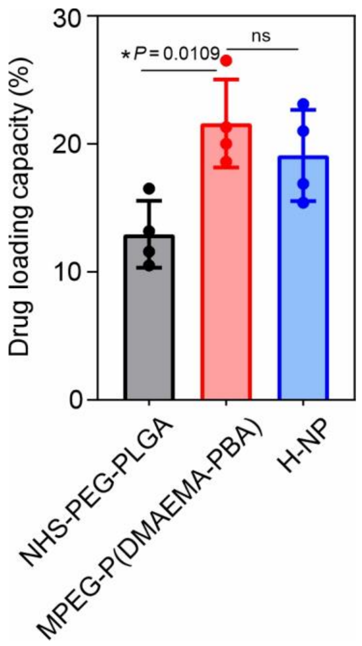

Supplementary Figure 3. The drug loading capacity of NHS-PEG-PLGA nanoparticles, MPEG-P(DMAEMA-PBA) nanoparticles, and H-NPs. Data represent the mean ± s.d. (n = 4 independent samples). The data were analyzed by one-way two-sided ANOVA.

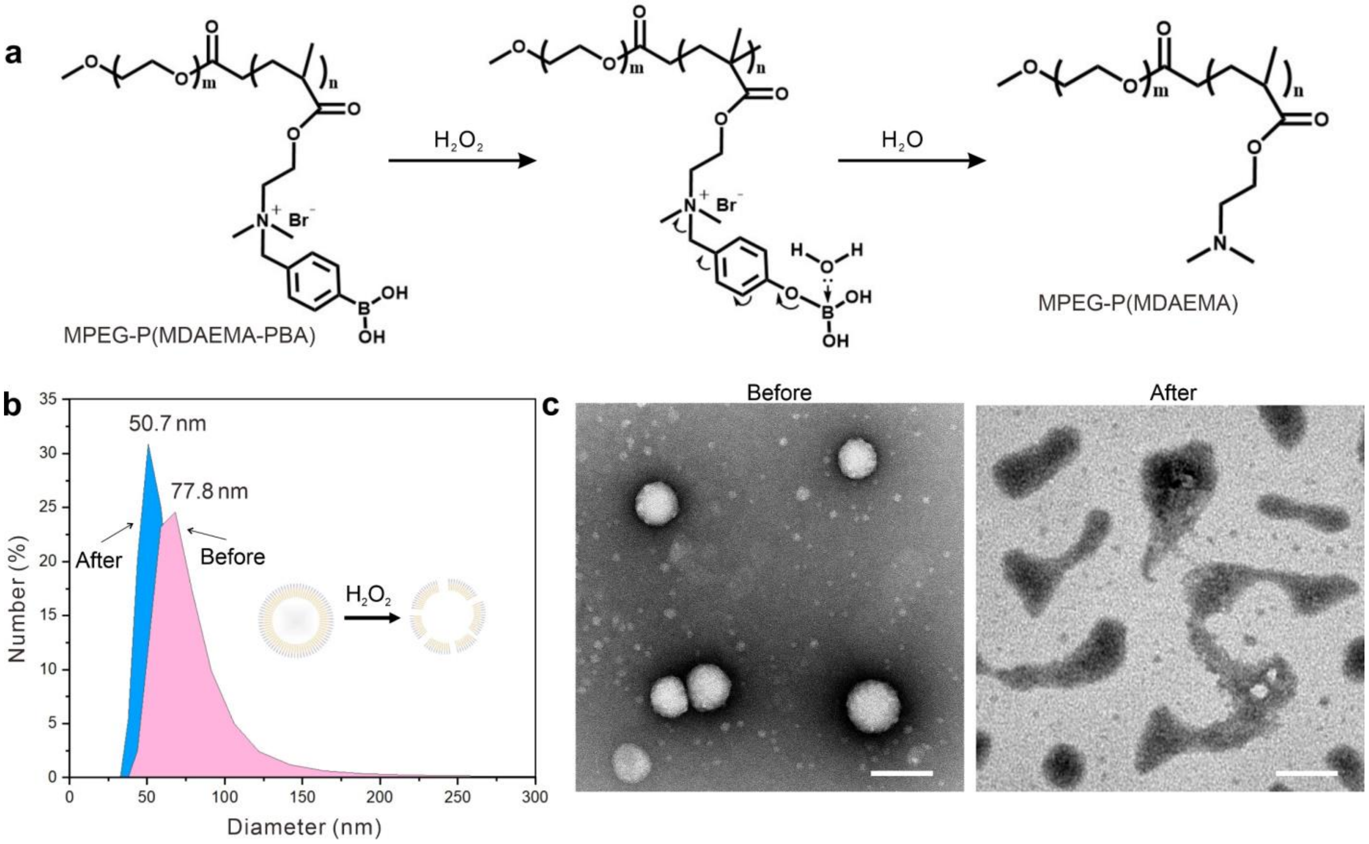

Supplementary Figure 4. ROS-responsive degradation behavior of H-NPs. a, The degradation mechanism of H-NPs. **b**, The diameter variations of H-NPs after the introduction of H_2O_2 (1 mM) were determined by DLS measurement. **c**, TEM images of H-NPs before and after the incubation in H_2O_2 solution (1 mM). Scale bar: 100 nm.

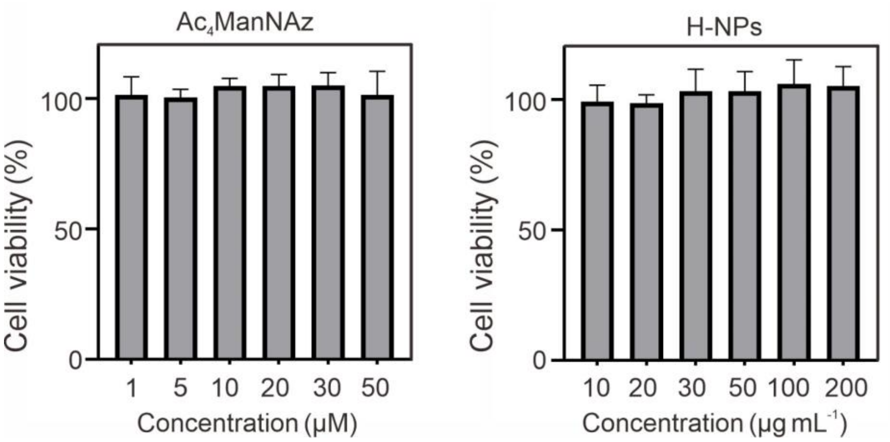

Supplementary Figure 5. The cytotoxicity of $Ac_4ManNAz$ and H-NPs with different concentrations. Data represent the mean ± s.d. (*n* = 4 independent samples).

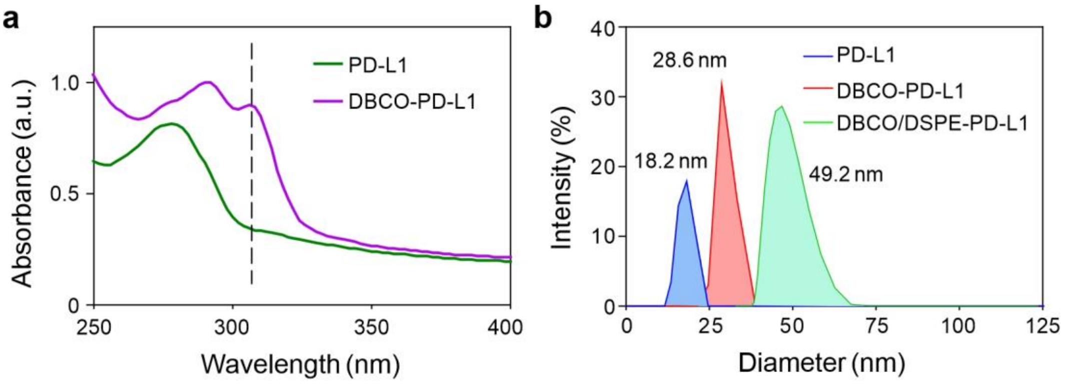

Supplementary Figure 6. The characterization of PD-L1 analogs. **a**, UV-visible absorption spectra of 0.5 mg mL^{-1} of PD-L1 and DBCO-PD-L1. **b**, The diameters of PD-L1, DBCO-PD-L1, and DBCO/DSPE-PD-L1 were determined by DLS measurement.

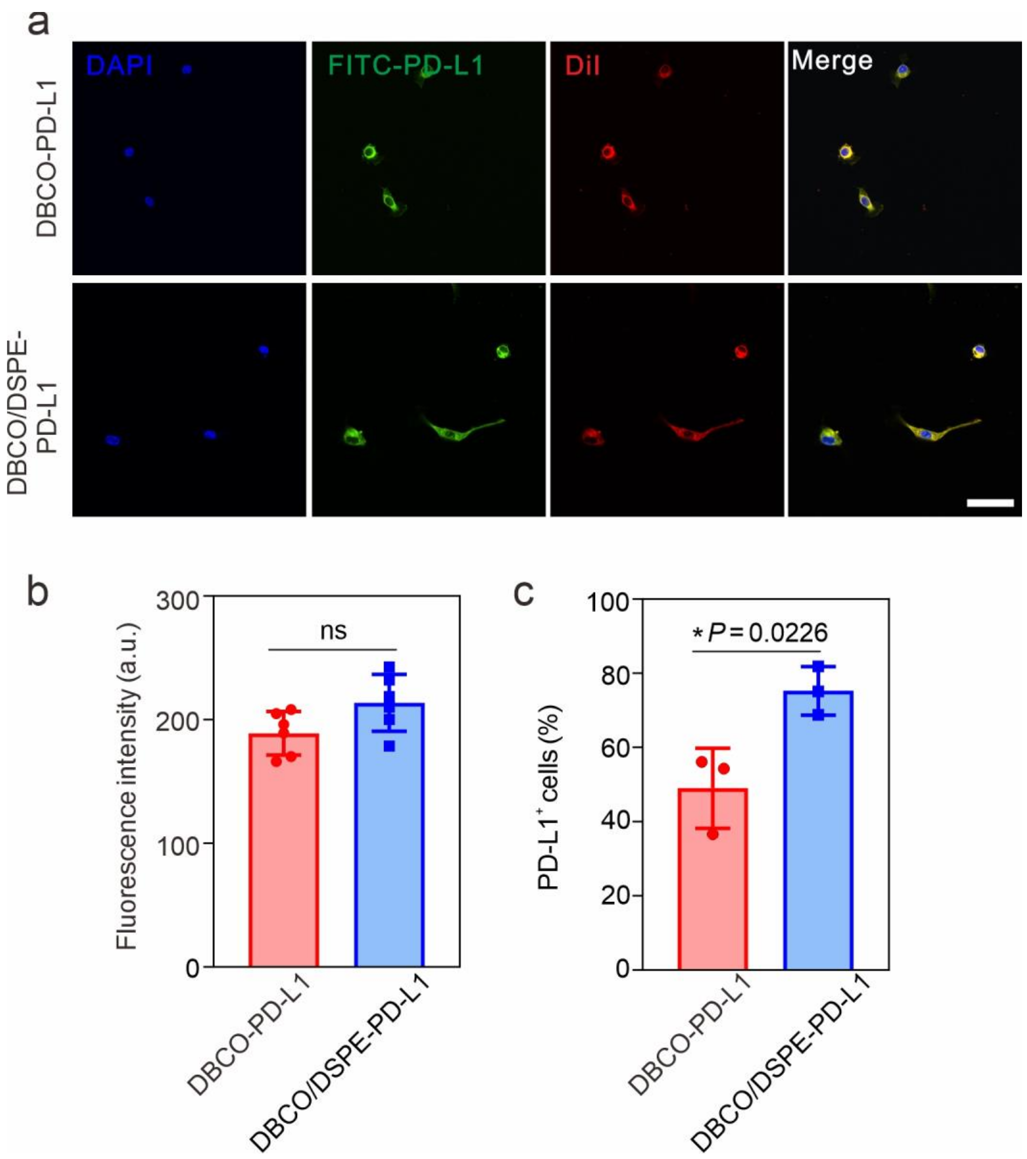

Supplementary Figure 7. The immobilization performance of PD-L1 analogs on the cell membrane. **a**,**b**, Confocal imaging (**a**) and quantitative assay (**b**) of Min 6 cells incubated with FITC-labeled PD-L1 analogs on day 1. Scale bar: 50 µm. Data represent the mean ± s.d. (n = 6 independent samples). **c**, Quantitative assay of Min 6 cells labeled with PD-L1 analogs on Day 3 *via* flow cytometry. Data represent the mean ± s.d. (n = 3 independent samples). The data were analyzed by two-tailed Student's t-test. $^{*}P<0.05$.

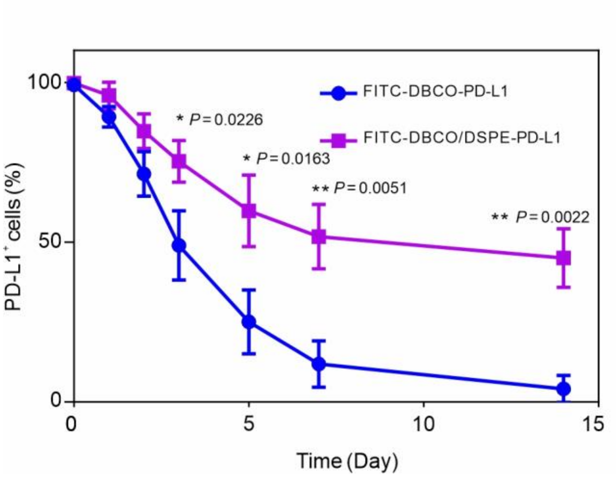

Supplementary Figure 8. The dual-anchor coupling strategy prolonged the immobilization of PD-L1 on the cell membrane. Quantitative assay of Min 6 cells incubating with FITC-labeled PD-L1 analogs. Data represent the mean ± s.d. (n = 3 independent samples). The data were analyzed by two-tailed Student's t-test. $^{**}P < 0.01$, $^{*}P < 0.05$.

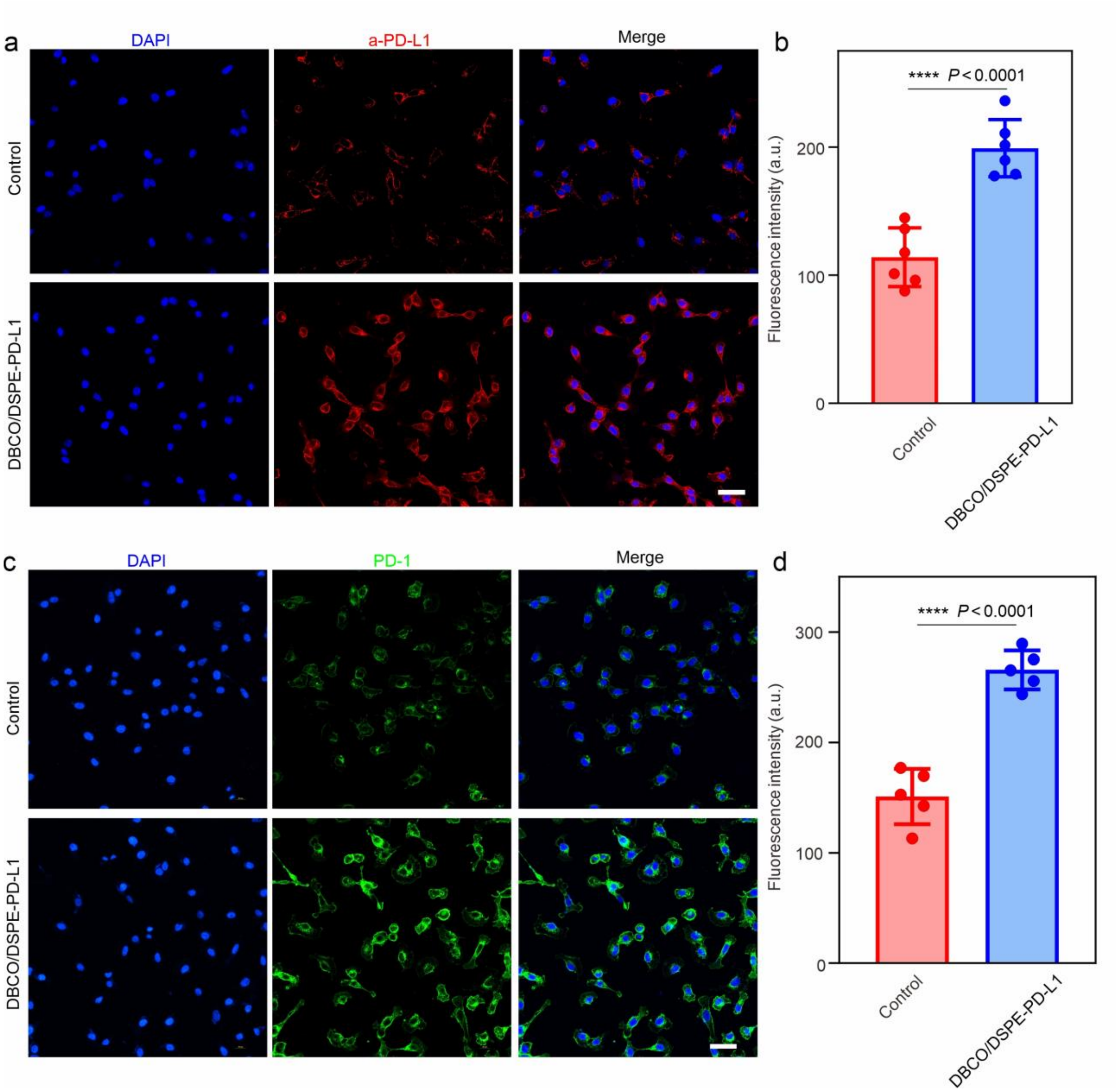

Supplementary Figure 9. **The dual-anchor coupling strategy enhanced the expression of PD-L1 on Min 6 cells. a**,**b**, Confocal imaging (**a**) and quantitative assay (**b**) of Min 6 cells stained with a-PD-L1 (red). Scale bar: 50 μm. Data represent the mean ± s.d. (*n* = 6 independent samples). **c**,**d**, Confocal imaging (c) and quantitative assay (d) of Min 6 cells stained with PD-1 (green). Scale bar: 50 μm. Data represent the mean ± s.d. (*n* = 5 independent samples). The data were analyzed by two-tailed Student's *t*-test. $^{****}P<0.0001$.

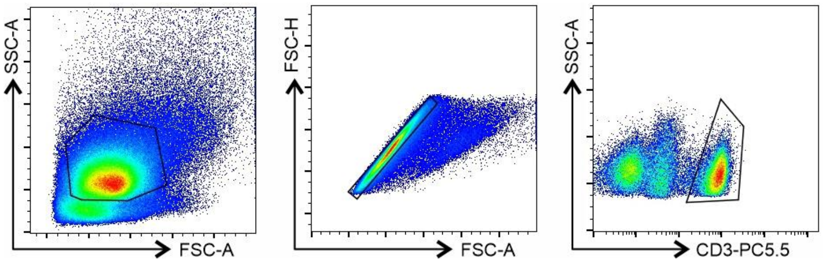

Supplementary Figure 10. Gating strategy for T cells analysis by flow cytometry in the *in vitro* experiment.

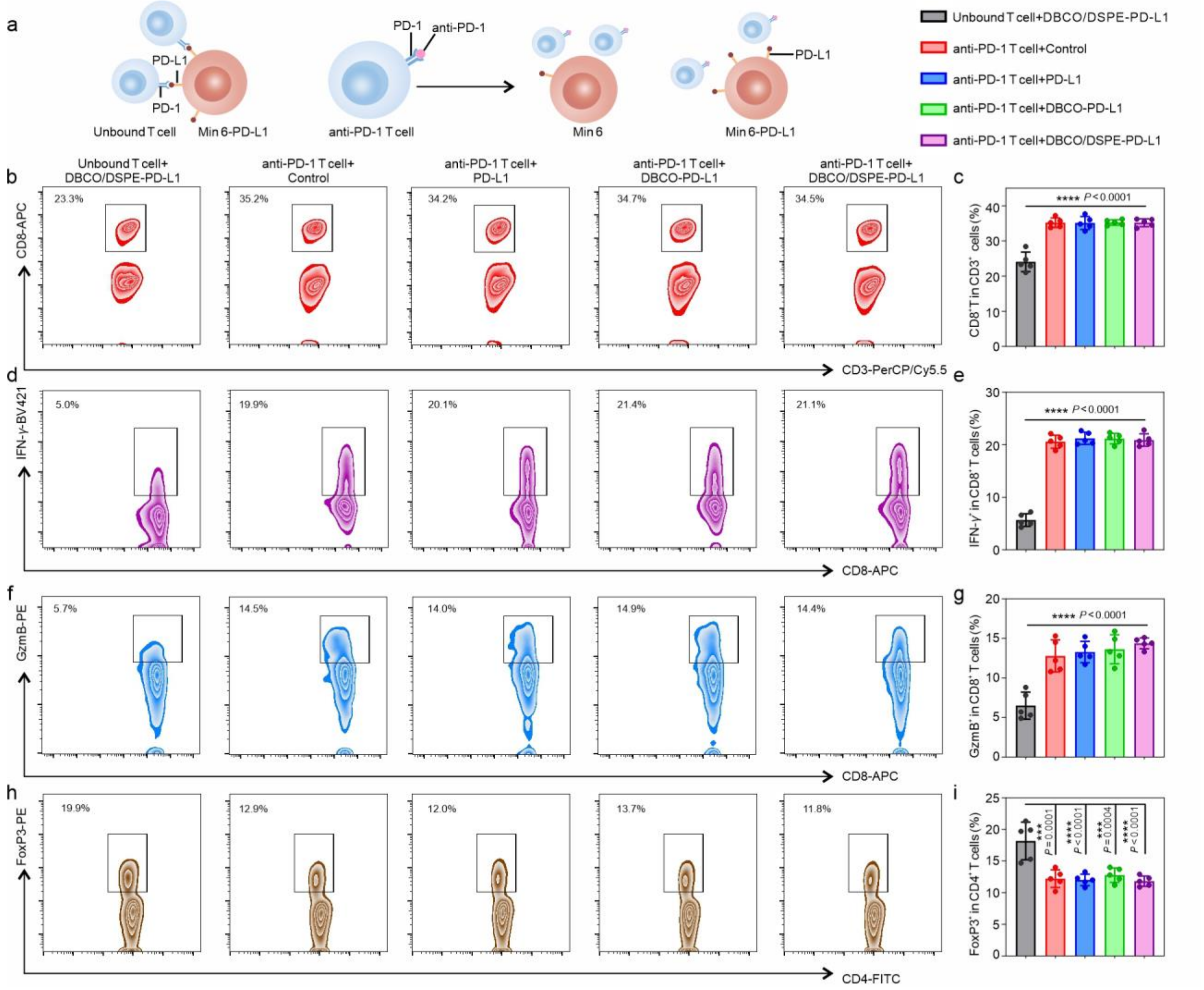

Supplementary Figure 11. Characterizations of the status of anti-PD-1-treated T cells in different treatment groups analyzed by flow cytometry. a, Schematic illustration revealed that the introduction of anti-PD-1 blocked the interactions between T cells and Min 6 cells. **b**,**c**, Representative plots (**a**) and quantification (**b**) of $CD8^+$ T cells in different treatment groups analyzed by flow cytometry. **d**,**e**, Representative plots (**d**) and quantifications (**e**) of $CD8^+INF\text{-}\gamma^+$ T cells in different treatment groups analyzed by the flow cytometry. **f**,**g**, Representative plots (**f**) and quantifications (**g**) of $CD8^+GzmB^+$ T cells in different treatment groups analyzed by flow cytometry. **h**,**i**, Representative plots (**h**) and quantification (**i**) of $CD4^+FoxP3^+$ T cells in different treatment groups analyzed by flow cytometry. Data represent the mean ± s.d. (*n* = 5 biologically independent samples). The data were analyzed by one-way two-sided ANOVA. $^{***}P$<0.001, $^{****}P$<0.0001.

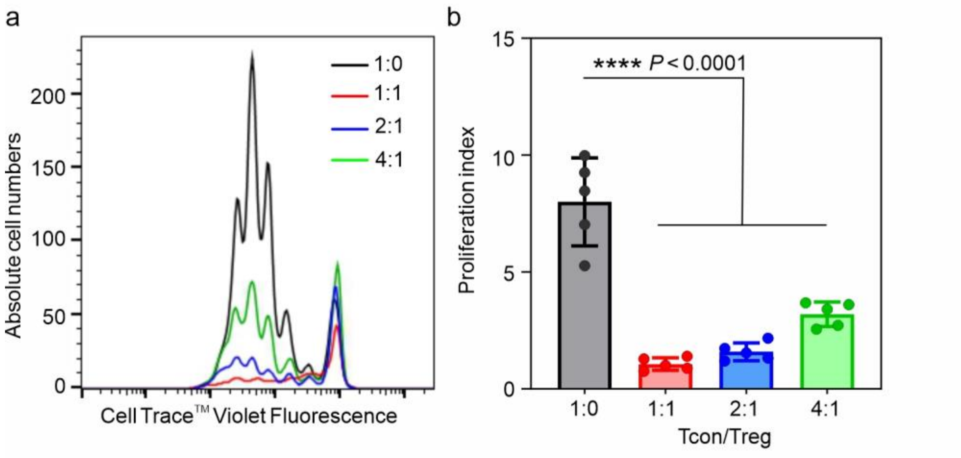

Supplementary Figure 12. Treg cell suppressive activity. a,**b**, Suppressive activity (**a**) and proliferation index (**b**) of purified Treg cells co-cultured with CellTrace™ stained Tcon cells was measured at day 5. Data represent the mean ± s.d. (n = 5 independent samples). The data were analyzed by one-way two-sided ANOVA. $^{****}P<0.0001$.

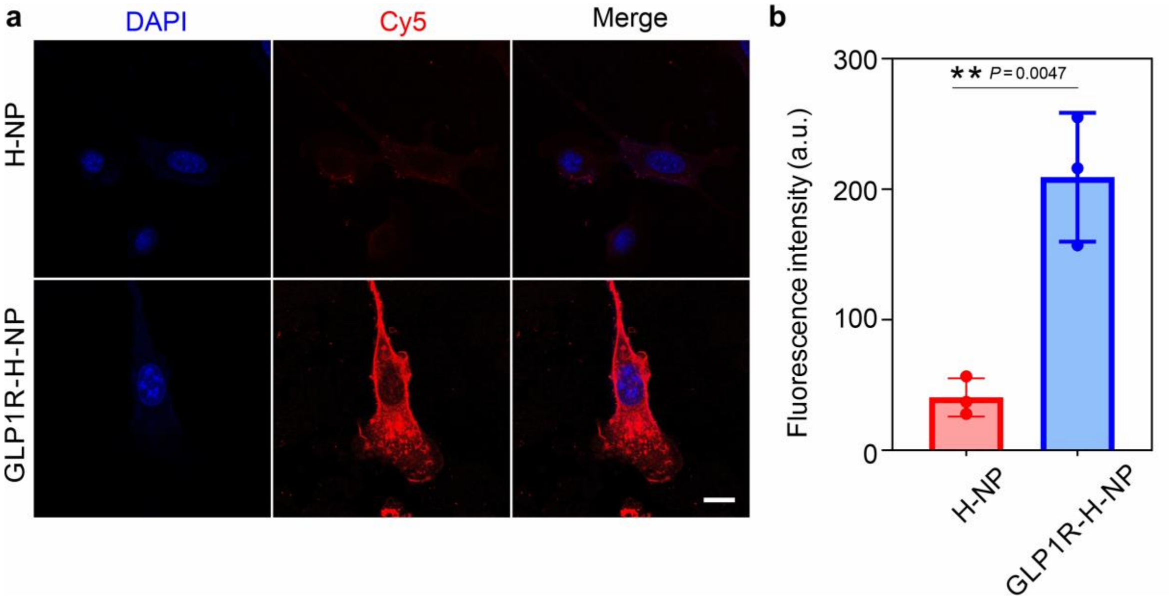

Supplementary Figure 13. GLP1R-H-NPs could be preferably internalized into pancreatic cells. a,**b**, Confocal imaging (**a**) and quantitative assay (**b**) of Min 6 cells incubating with Cy5-labeled H-NPs and GLP1R-H-NPs. Scale bar: 10 μm. Data represent the mean ± s.d. (n = 3 independent samples). The data were analyzed by a two-tailed Student's t-test. $^{**}P < 0.01$.

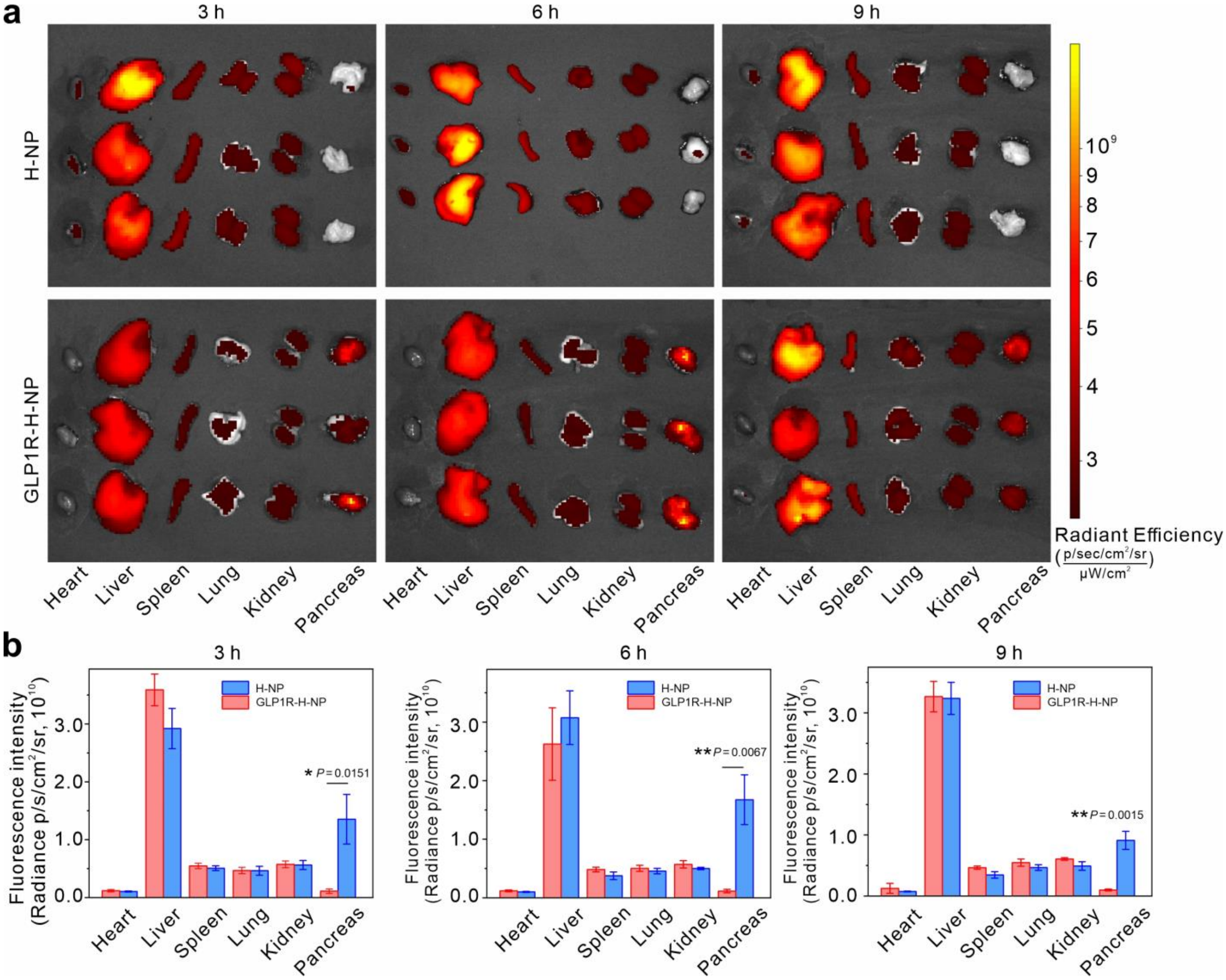

Supplementary Figure 14. GLP1R facilitated H-NPs delivery to the pancreas of NOD mice. a, *Ex vivo* images revealed the biodistribution of H-NPs and GLP1R-H-NPs at predetermined time points. **b**, Quantifications of fluorescent signals in the main organs. Data represent the mean ± s.d. (n = 3 biologically independent samples). The data were analyzed by a two-tailed Student's t-test; $^{***}P<0.001$.

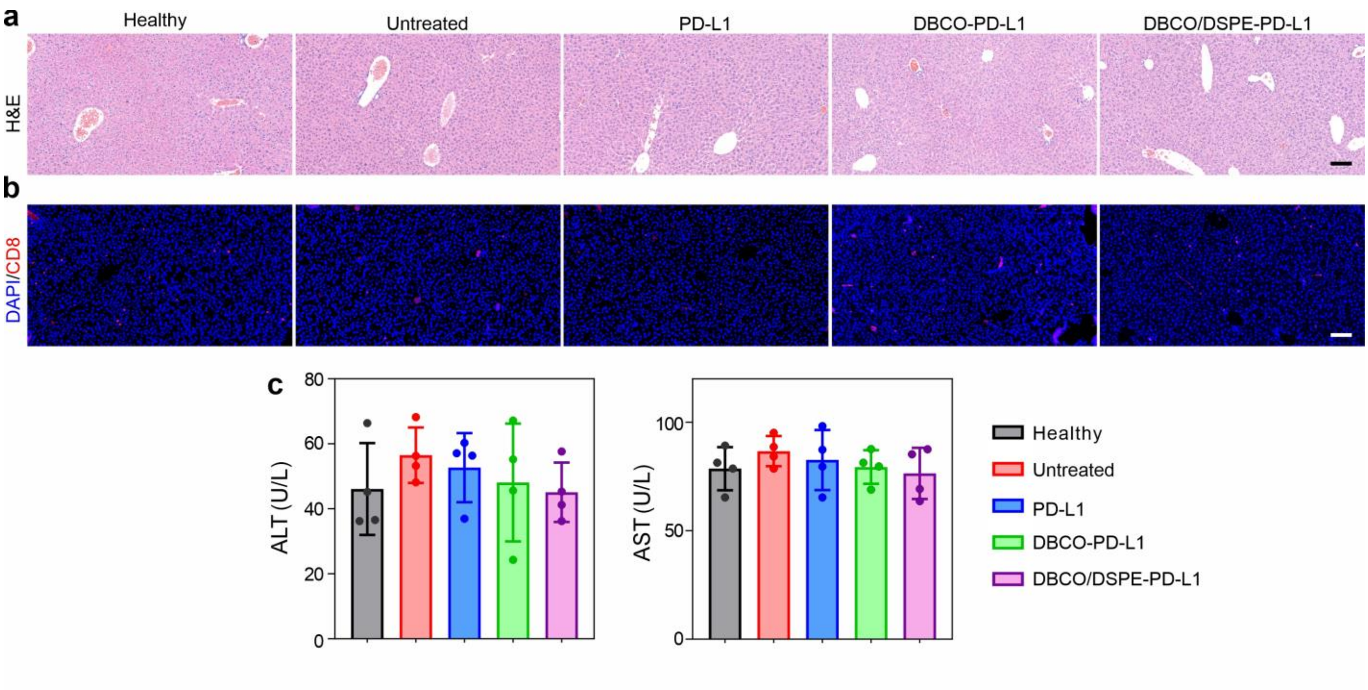

Supplementary Figure 15. Histological analysis of liver. a, Representative H&E staining of liver sections from NOD mice after different treatments. Scale bar: 100 µm. **b**, Representative anti-CD8 (red) stained liver sections from NOD mice after different treatments. Scale bar: 100 µm. **c**, Blood biochemistry analysis of NOD mice after different treatments. AST: aspartate transferase, ALT: alanine transferase. Data are presented as mean ± s.d. (n = 4 biologically independent samples).

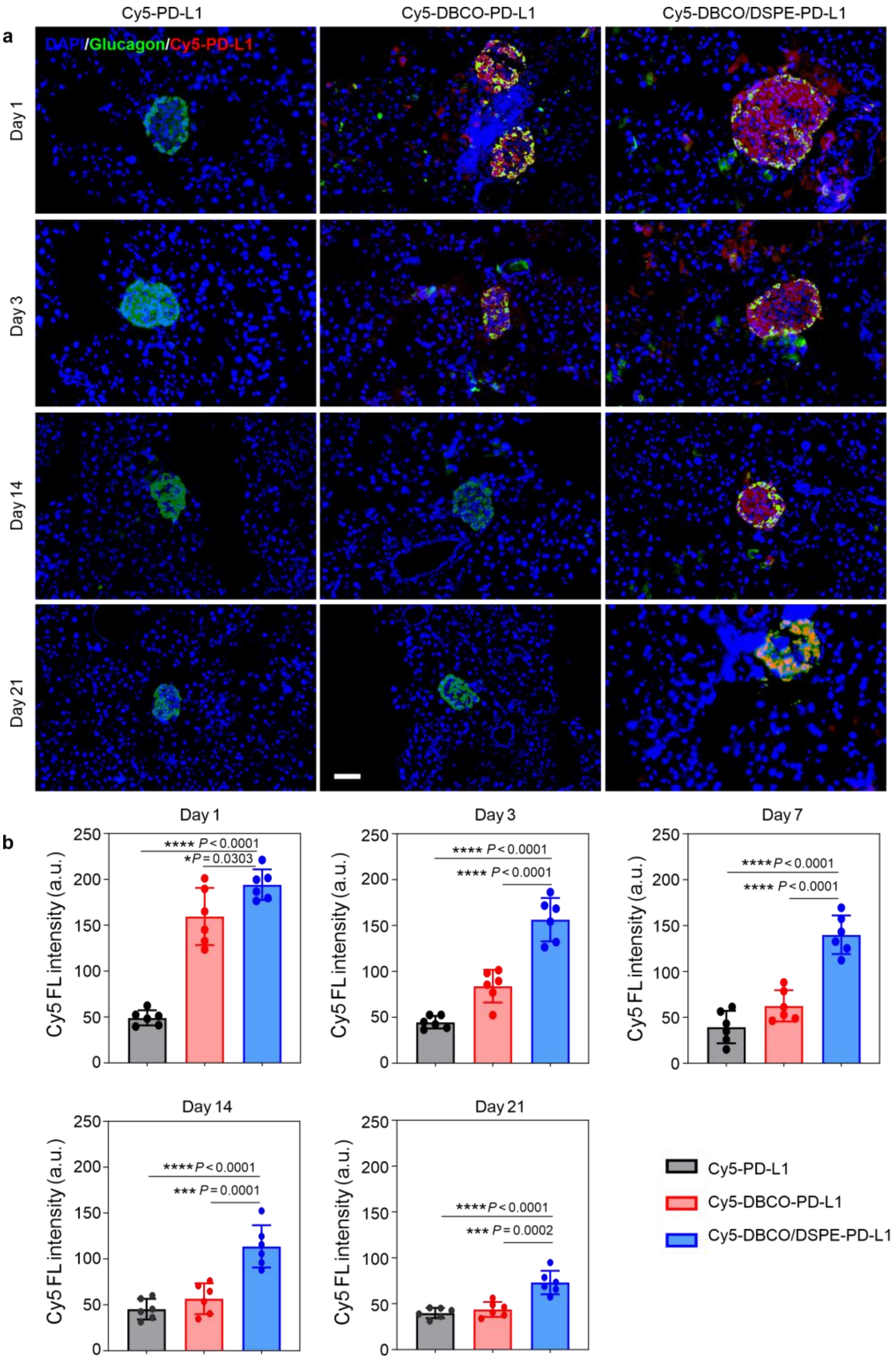

Supplementary Figure 16. The dual-anchor coupling strategy prolonged the immobilization of PD-L1 on islets. a,b, Fluorescent imaging (**a**) and quantifications (**b**) of fluorescent signals of the pancreas from NOD mice *i.v.* injected with Cy5-labeled PD-L1 analogs at different time points. Scale bar: 50 μm. Data represent the mean ± s.d. (n = 6 biologically independent samples). The data were analyzed by one-way two-sided ANOVA. $^{****}P$<0.0001, $^{***}P$<0.001.

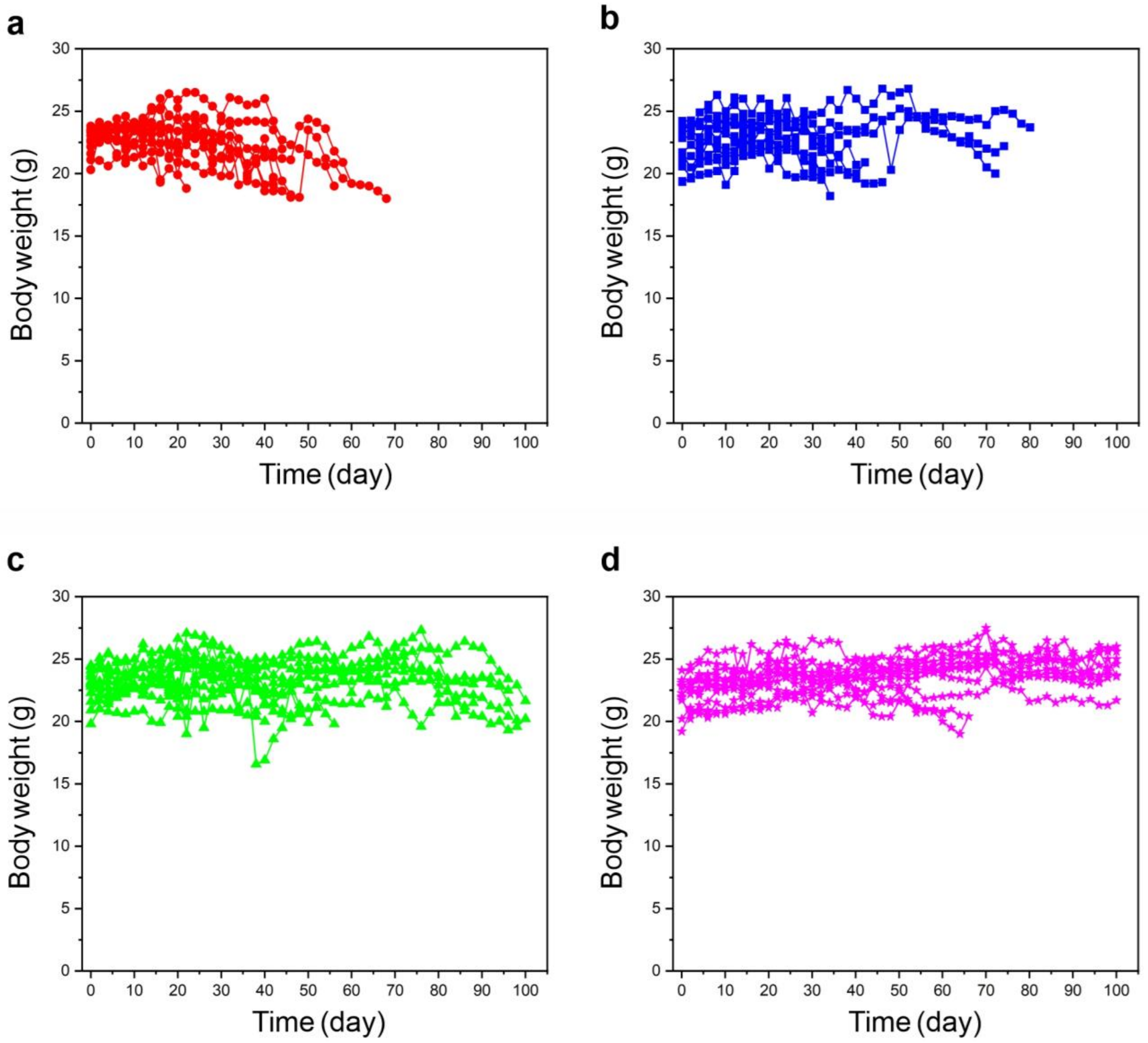

Supplementary Figure 17. Body weights of the diabetic NOD mice with different treatments. a-d, The body weights of NOD mice treated with untreated group (a), PD-L1 group (b), DBCO-PD-L1 (c), and DBCO/DSPE-PD-L1 group (d).

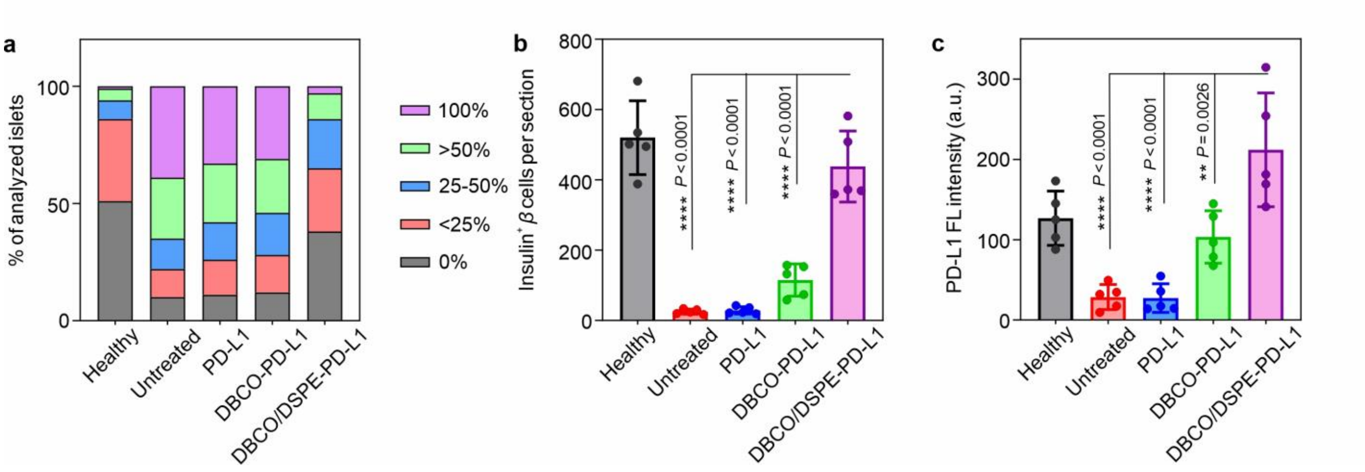

Supplementary Figure 18. PD-L1 bioengineering reverses the early-onset type 1 diabetes in the NOD mice. a, Insulitis scores of pancreatic sections. **b**, Quantification of insulin^{+} β-cells in the pancreas sections. **c**, Quantification of the fluorescence intensities of PD-L1 in the pancreas sections. Data represent the mean ± s.d. (*n* = 5 biologically independent samples). The data were analyzed by one-way two-sided ANOVA. $^{****}P<0.0001$, $^{**}P< 0.01$.

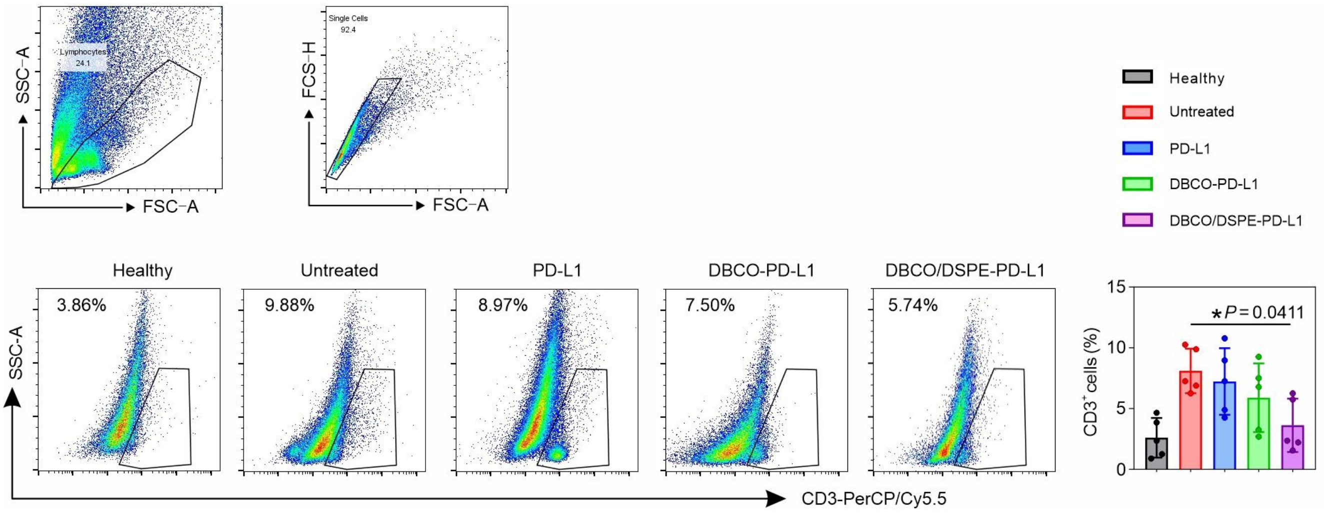

Supplementary Figure 19. Representative plots and quantification of pancreas-infiltrating CD3^{+} T cells in different treatment groups analyzed by flow cytometry. Data represent the mean ± s.d. (n = 5 biologically independent samples). The data were analyzed by one-way two-sided ANOVA; $^{*}P$<0.05.

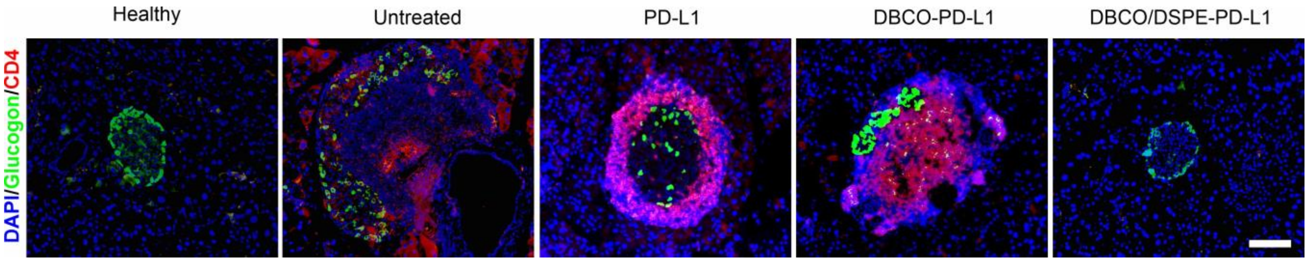

Supplementary Figure 20. Characterization of CD4$^+$ T cells in the pancreas of NOD mice. Representative anti-CD4 (red)/anti-glucagon (green) dual stained pancreas sections from NOD mice on day 5 post-treatment. Scale bar: 100 µm.

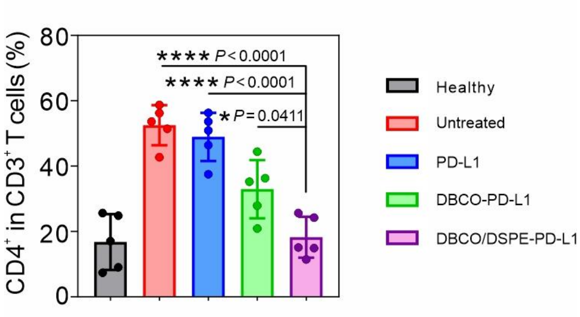

Supplementary Figure 21. Quantification of pancreas-infiltrating CD4$^+$ T cells. Data represent the mean ± s.d. (*n* = 5 biologically independent samples). The data were analyzed by one-way two-sided ANOVA. $^{****}P<0.0001$, $^{*}P<0.05$.

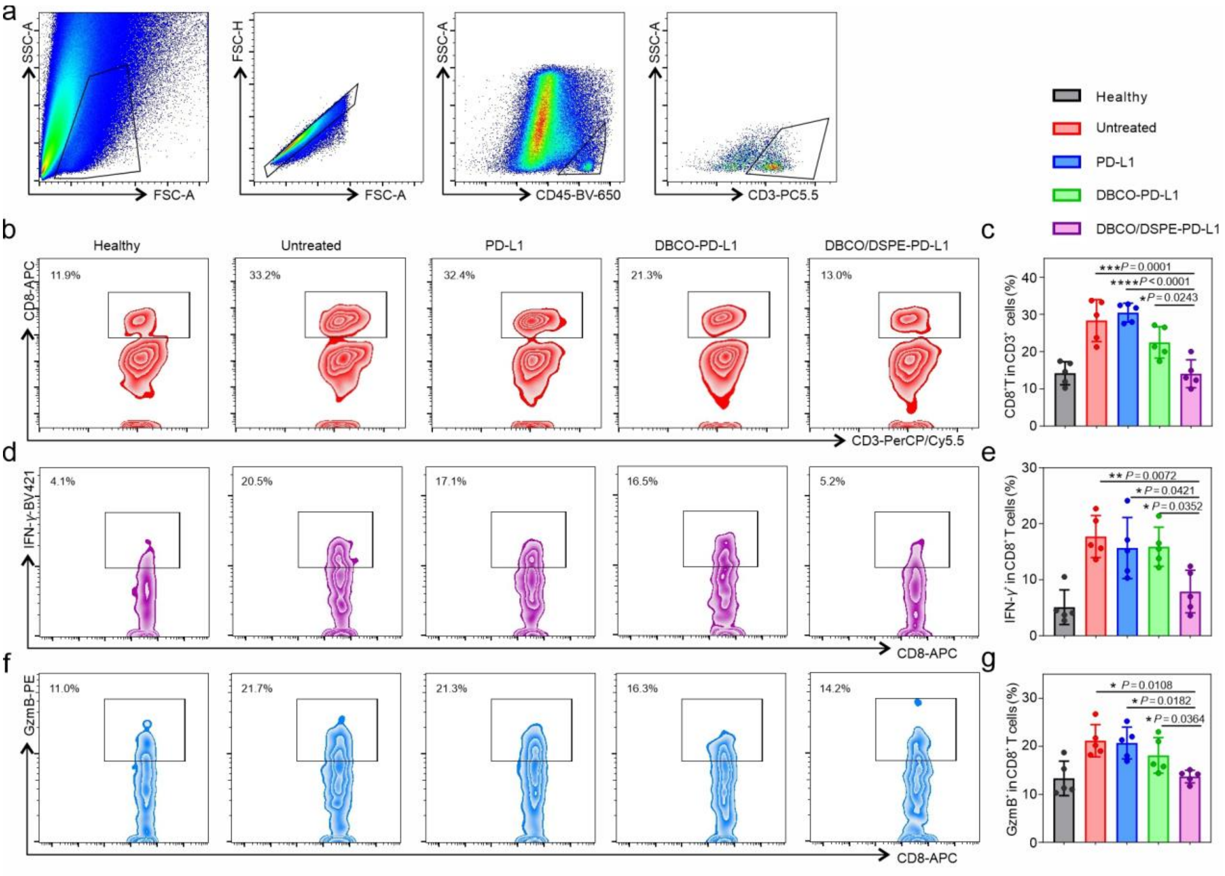

Supplementary Figure 22. Characterizations of the T-cell status in the pancreas of diabetic NOD mice. **a**, Gating strategies for T cells analysis by flow cytometry in the pancreas on day 10 post-treatments. **b**,**c**, Representative plots of pancreas-infiltrating $CD4^+$ and $CD8^+$ T cells (**b**) and quantification of pancreas-infiltrating $CD8^+$ T cells (**c**). **d**,**e**, Representative plots (**d**) and quantifications (**e**) of pancreas-infiltrating $CD8^+INF\text{-}\gamma^+$ T cells in different treatment groups analyzed by the flow cytometry. **f**,**g**, Representative plots (**f**) and quantifications (**g**) of pancreas-infiltrating $CD8^+GzmB^+$ T cells in different treatment groups analyzed by the flow cytometry. Data represent the mean ± s.d. (n = 5 biologically independent samples). The data were analyzed by one-way two-sided ANOVA. $^{****}P<0.0001$, $^{***}P<0.001$, $^{**}P<0.01$, $^{*}P<0.05$.

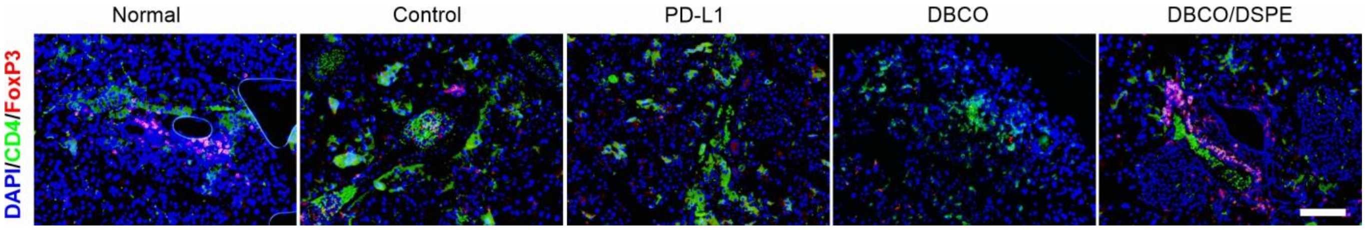

Supplementary Figure 23. Characterization of Treg cells in the pancreas of NOD mice. Representative anti-CD4 (green)/anti-FoxP3 (red) dual-stained pancreas sections from NOD mice on day 5 post-treatments. Scale bar: 100 μm.

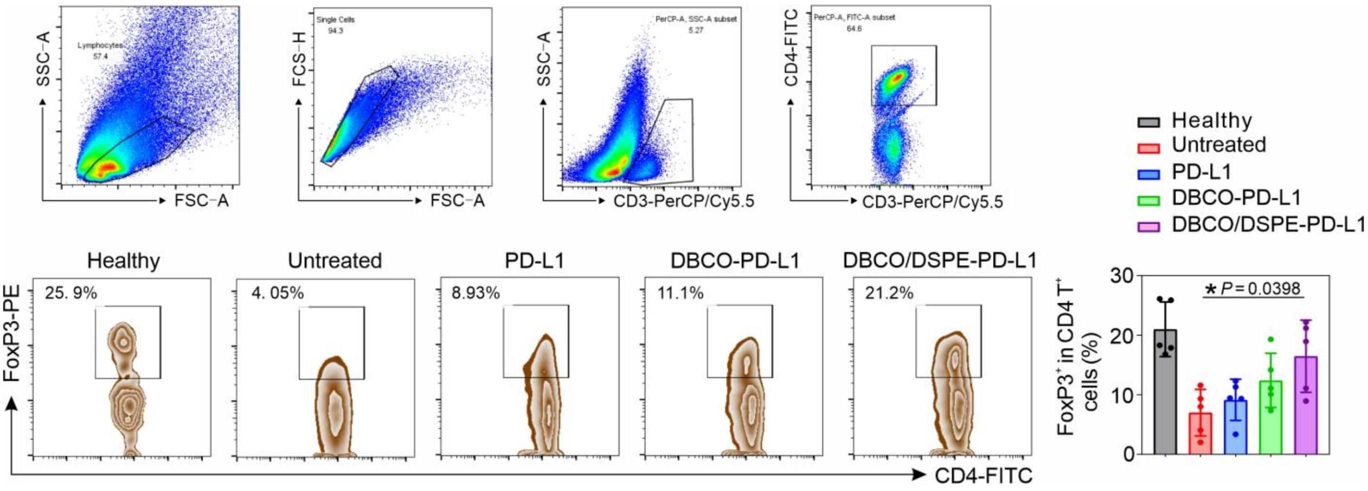

Supplementary Figure 24. Representative plots and quantification of pancreas-infiltrating FoxP3^{+} T cells in different treatment groups analyzed by flow cytometry. Data represent the mean ± s.d. (n = 5 biologically independent samples). The data were analyzed by one-way two-sided ANOVA. $^{*}P$<0.05.

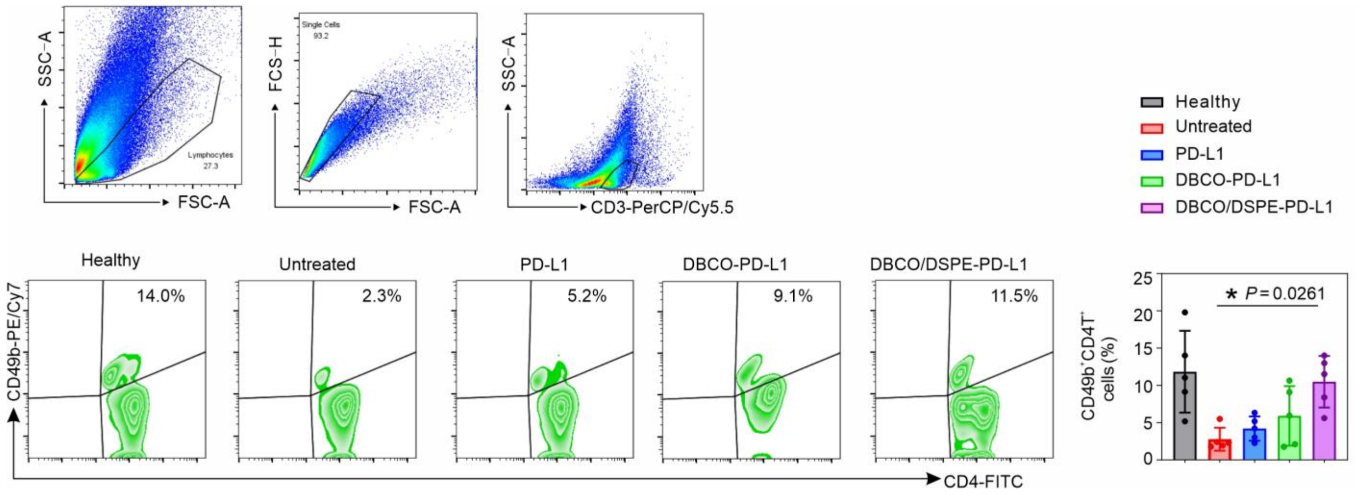

Supplementary Figure 25. Representative plots and quantification of pancreas-infiltrating CD4⁺CD49b⁺ T cells in different treatment groups analyzed by flow cytometry. Data represent the mean ± s.d. (*n* = 5 biologically independent samples). The data were analyzed by one-way two-sided ANOVA. $^{*}P$<0.05.

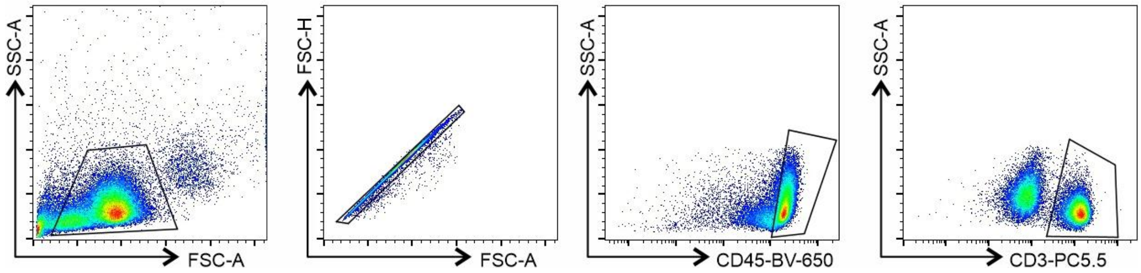

Supplementary Figure 26. Gating strategy for T cells analysis by flow cytometry in the lymph node (LN) of NOD mice.

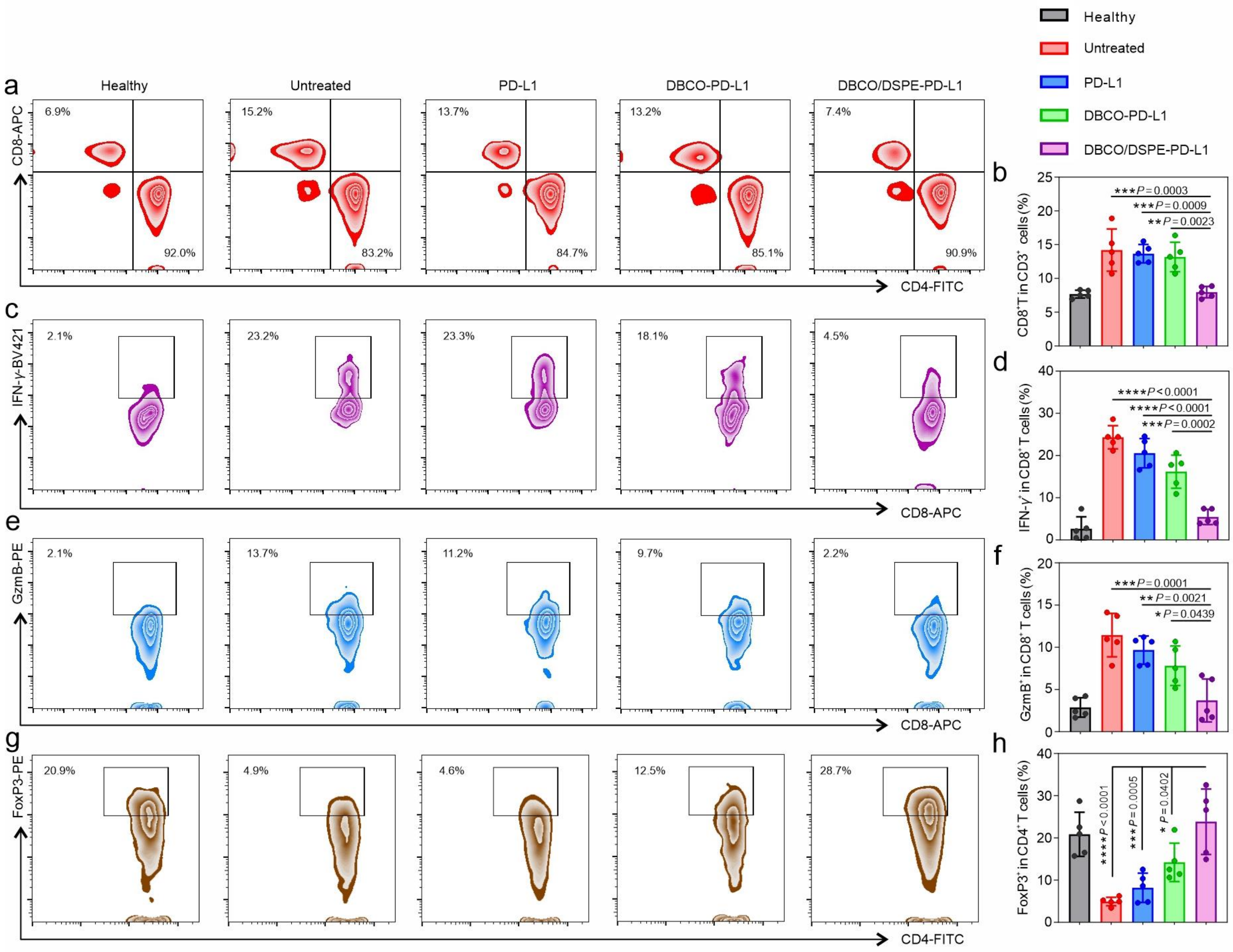

Supplementary Figure 27. Characterizations of the T-cell status in the lymph node (LN) of diabetic NOD mice. a,**b**, Representative plots of LN-infiltrating CD4$^+$ and CD8$^+$ T cells (**a**) and quantification of LN-infiltrating CD8$^+$ T cells (**b**). **c**,**d**, Representative plots (**c**) and quantifications (**d**) of LN-infiltrating CD8$^+$INF-γ^+ T cells in different treatment groups analyzed by the flow cytometry. **e**,**f**, Representative plots (**e**) and quantifications (**f**) of LN-infiltrating CD8$^+$GzmB$^+$ T cells in different treatment groups analyzed by the flow cytometry. **g**,**h**, Representative plots (**g**) and quantification (**h**) of LN-infiltrating FoxP3$^+$ T cells in different treatment groups analyzed by flow cytometry. Data represent the mean ± s.d. (n = 5 biologically independent samples). The data were analyzed by one-way two-sided ANOVA. $^{****}P$<0.0001, $^{***}P$<0.001, $^{**}P$< 0.01, $^{*}P$<0.05.

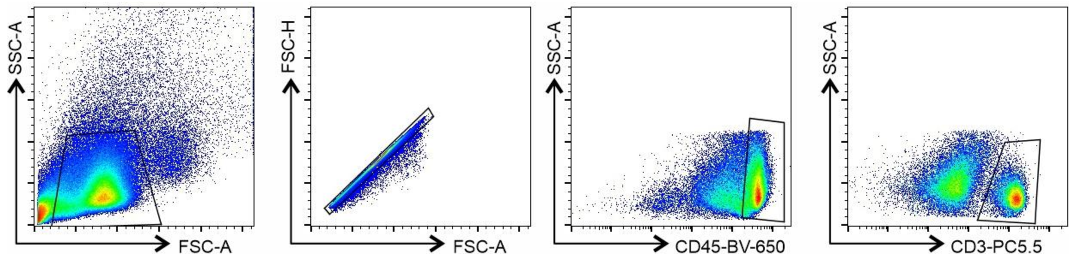

Supplementary Figure 28. Gating strategy for T cells analysis by flow cytometry in the spleen of NOD mice.

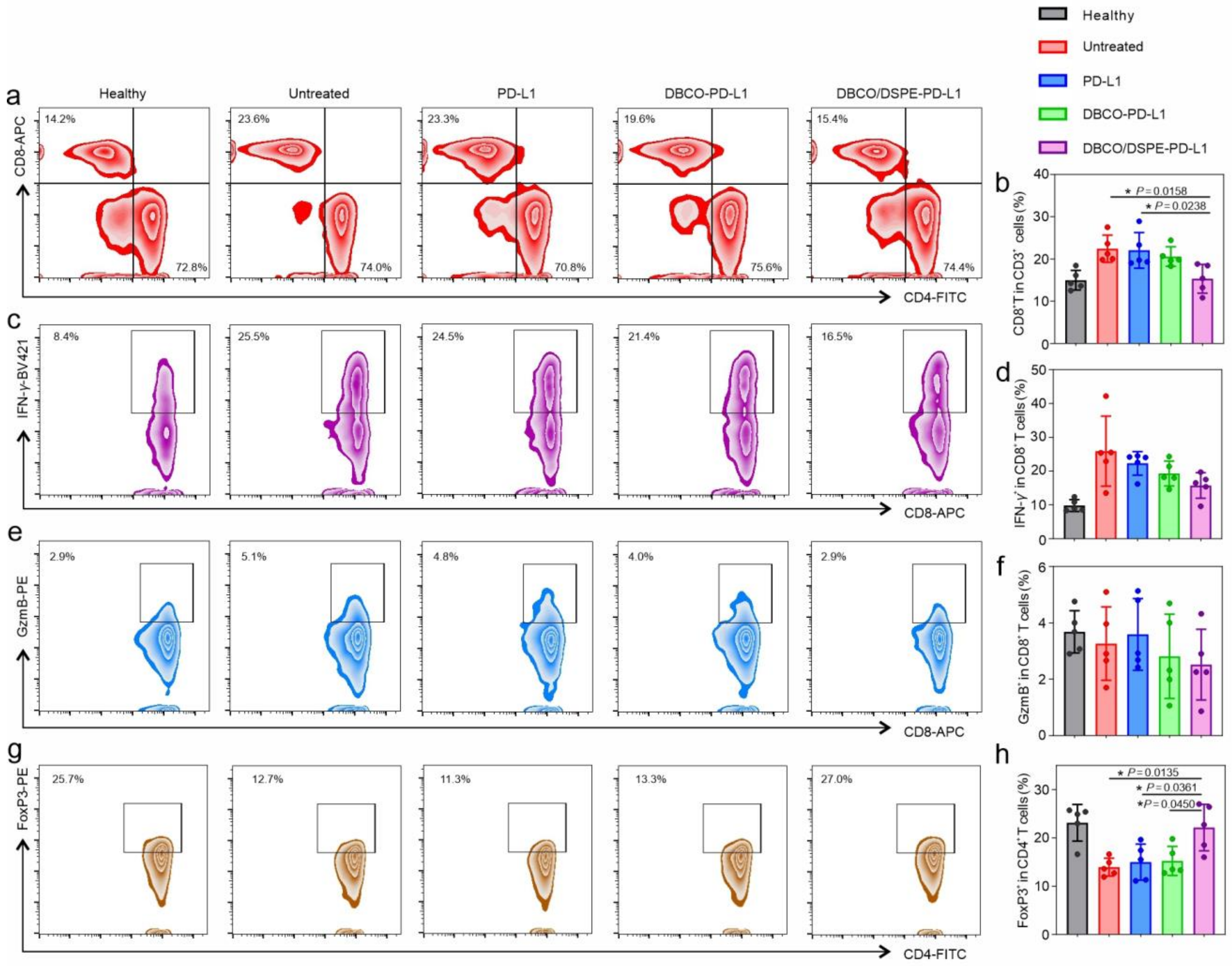

Supplementary Figure 29. Characterizations of the T-cell status in the spleen of diabetic NOD mice. **a**,**b**, Representative plots of spleen-infiltrating $CD4^+$ and $CD8^+$ T cells (**a**) and quantification of spleen-infiltrating $CD8^+$ T cells (**b**). **c**,**d**, Representative plots (**c**) and quantifications (**d**) of spleen-infiltrating $CD8^+INF\text{-}\gamma^+$ T cells in different treatment groups analyzed by the flow cytometry. **e**,**f**, Representative plots (**e**) and quantifications (**f**) of spleen-infiltrating $CD8^+GzmB^+$ T cells in different treatment groups analyzed by the flow cytometry. **g**,**h**, Representative plots (**g**) and quantification (**h**) of spleen-infiltrating $FoxP3^+$ T cells in different treatment groups analyzed by flow cytometry. Data represent the mean ± s.d. (n = 5 biologically independent samples). The data were analyzed by one-way two-sided ANOVA. $^*P<0.05$.

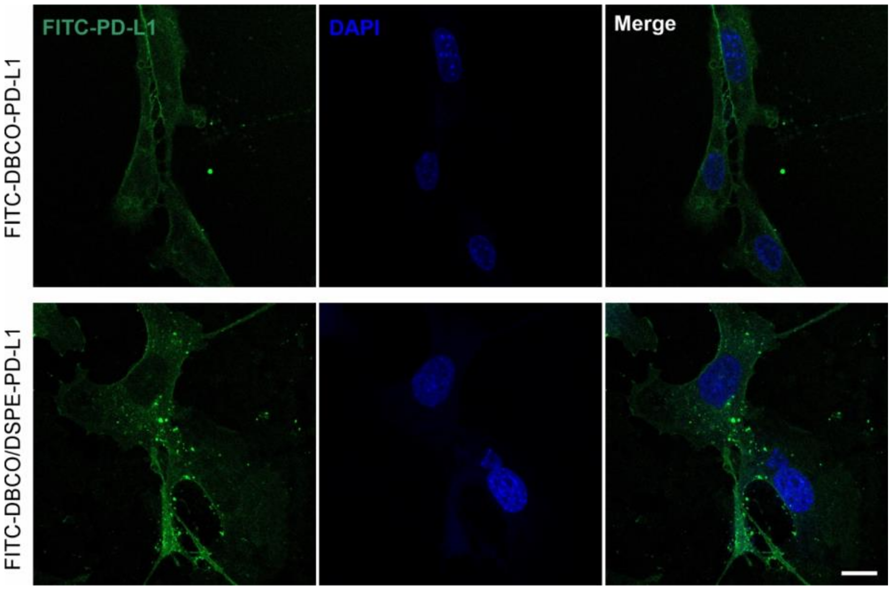

Supplementary Figure 30. Confocal imaging of chondrocytes labeled with FITC-labeled PD-L1 analogs on day 3. Scale bar: 20 μm.

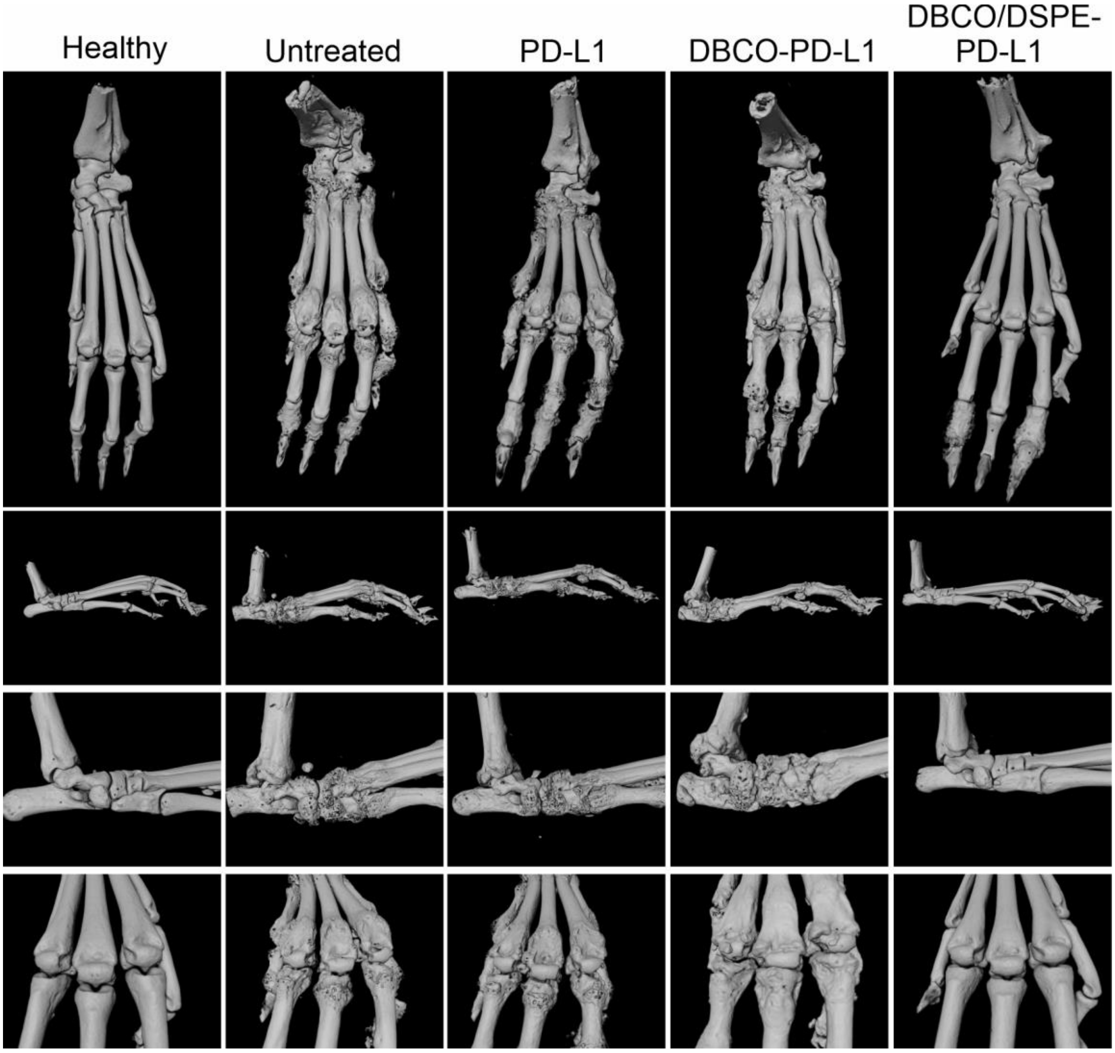

Supplementary Figure 31. Representative 3D-reconstructed micro-CT images of left hind ankle joints of normal and arthritic mice after different treatments on day 48.

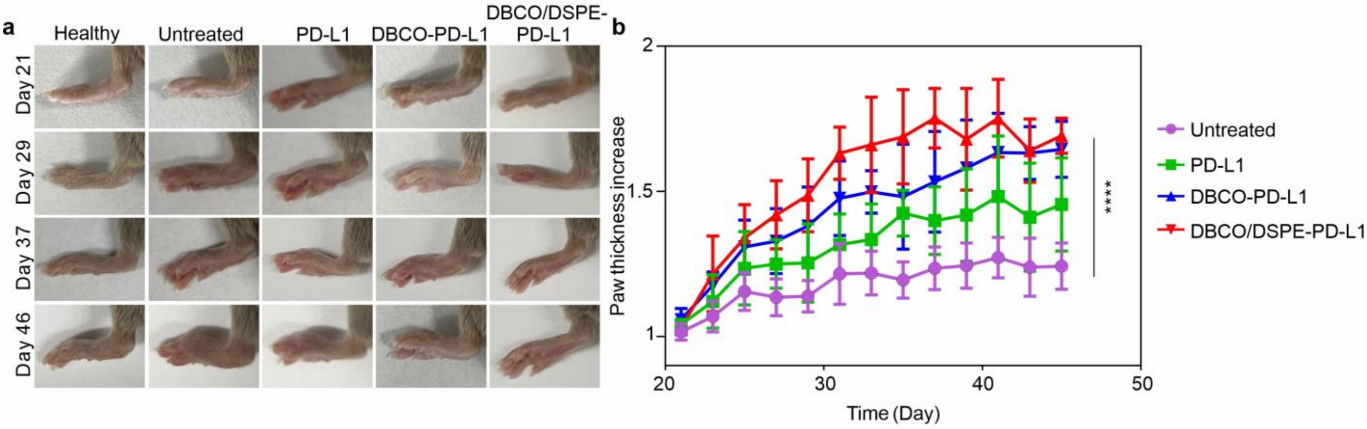

Supplementary Figure 32. *In situ* immobilization of PD-L1 ameliorates left joint swelling. a, Representative images of the left hind paw of mice in different treatment groups at the time of each treatment. **b**, Left hind ankle joint swelling behaviors of arthritic mice. Data represent the mean ± s.d. (*n* = 5 biologically independent samples). The data were analyzed by one-way two-sided ANOVA; $^{****}P<0.0001$.

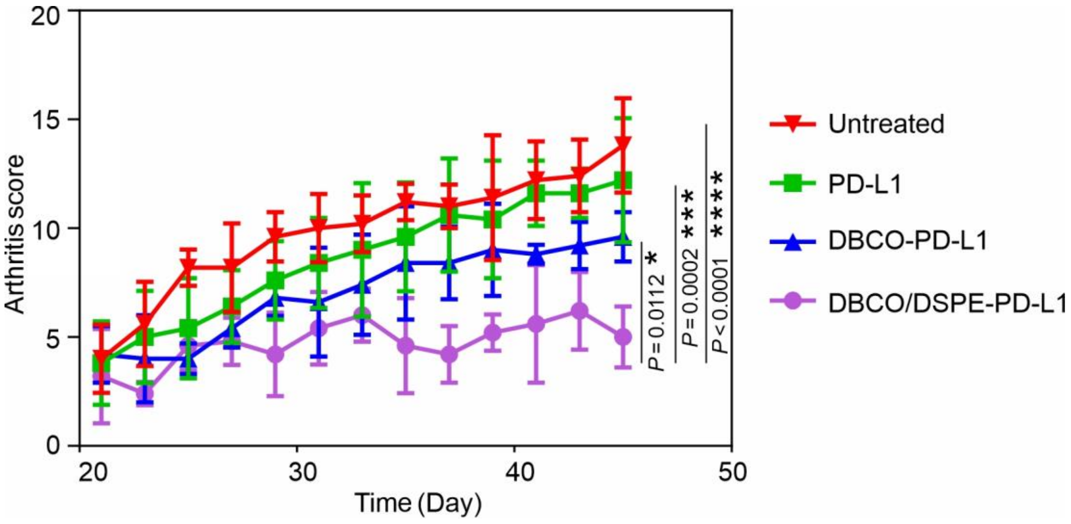

Supplementary Figure 33. The average Clinical score of arthritis in mice with different treatments. Data represent the mean ± s.d. (n = 5 biologically independent samples). The data were analyzed by one-way two-sided ANOVA. $^{****}P$<0.0001, $^{***}P$<0.001, $^{*}P$<0.05.

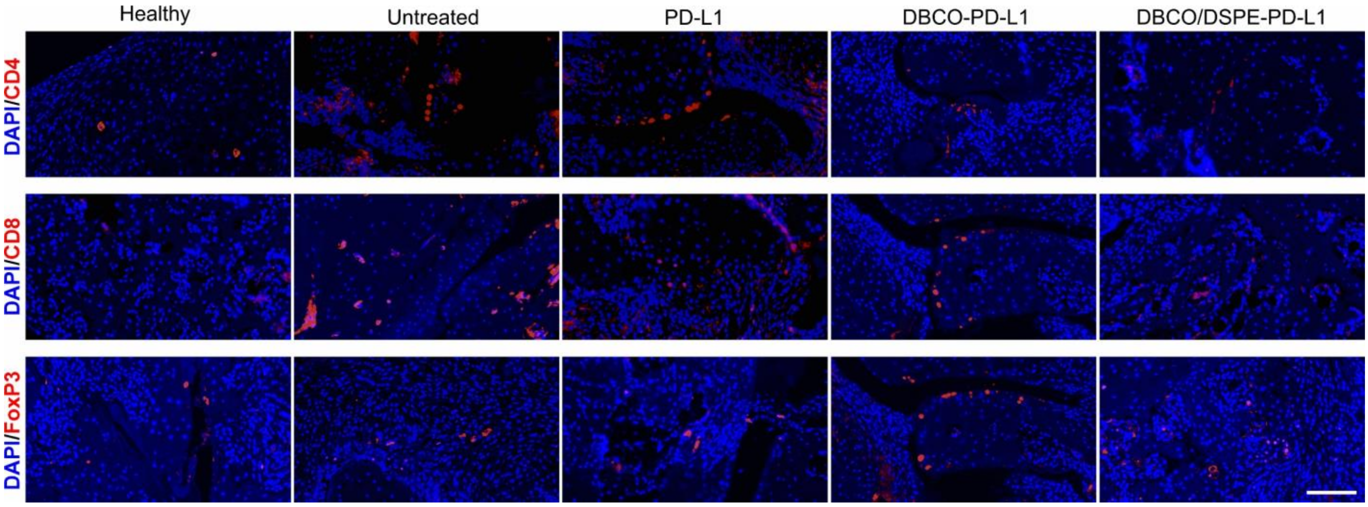

Supplementary Figure 34. Representative anti-CD4, anti-CD8, and anti-FoxP3 (red) stained knee sections from DBA mice after different treatments. Scale bar: 100 μm.

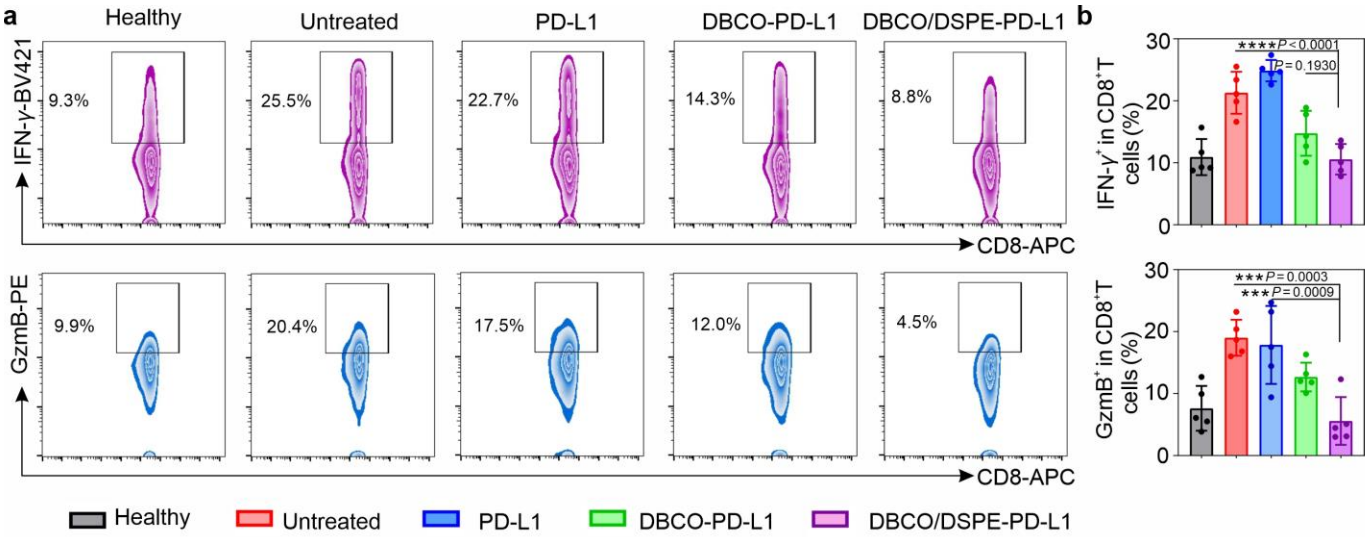

Supplementary Figure 35. The T cell status in the spleen of DBA mice. a,**b**, Representative plots (**a**) and quantifications (**b**) of CD8^{+}INF-γ^{+} T cells and CD8^{+}GzmB^{+} T cells in the spleen of DBA mice from different treatment groups were analyzed by flow cytometry. Data represent the mean ± s.d. (n = 5 biologically independent samples). The data were analyzed by one-way two-sided ANOVA. $^{****}P<0.0001$, $^{***}P<0.001$.

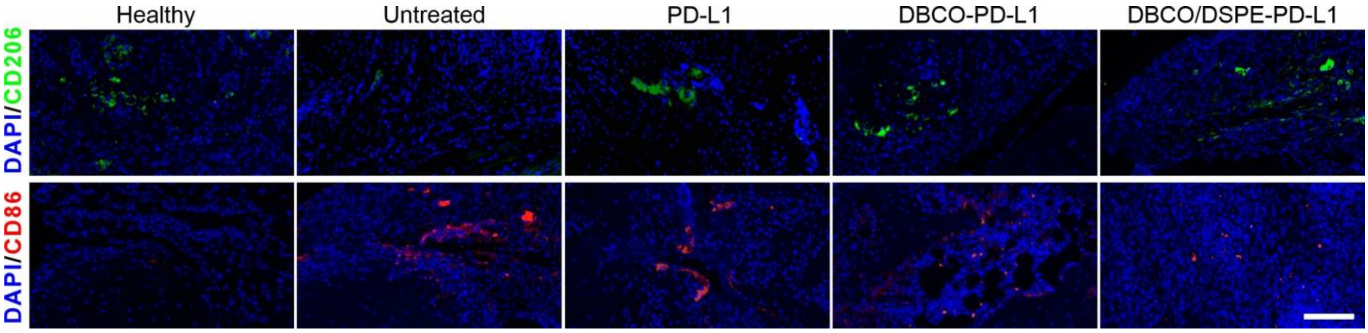

Supplementary Figure 36. Representative anti-CD206 (green) and anti-CD86 (red) stained knee sections from DBA mice after different treatments. Scale bar: 100 μm.

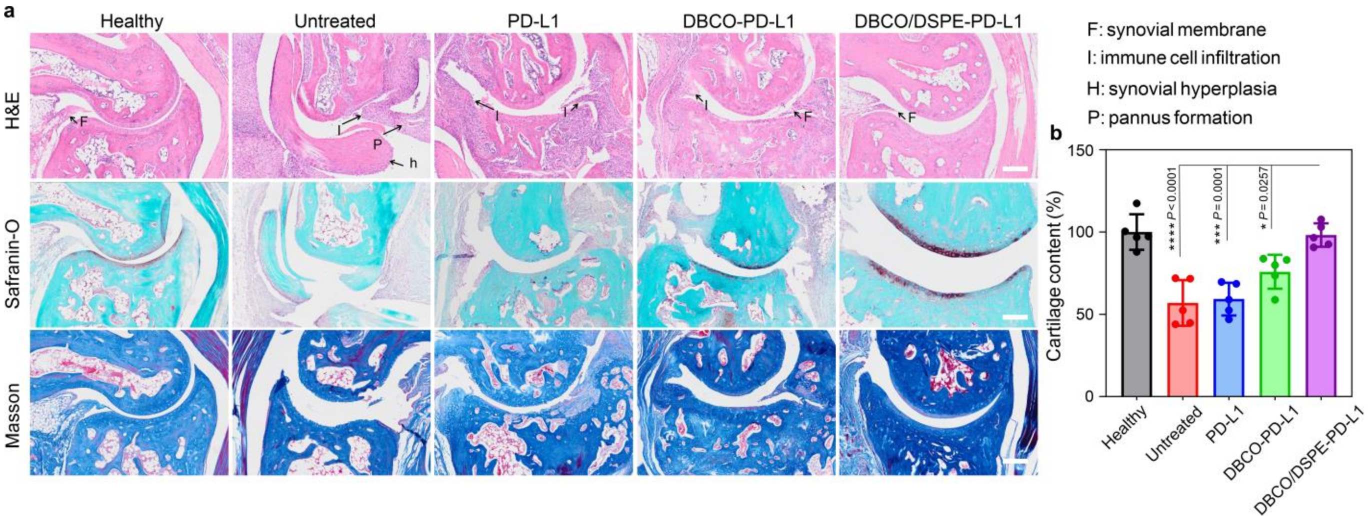

Supplementary Figure 37. ***In situ*** **immobilization of PD-L1 ameliorates joint disruption. a**, Representative images of H&E staining, safranin-O staining, and Masson staining on knee sections from mice with different treatments. **b**, Cartilage content quantified from safranin-O-stained sections of mice with different treatments. Scale bars: 200 μm. Data represent the mean ± s.d. (n = 5 biologically independent samples). The data were analyzed by one-way two-sided ANOVA. $^{****}P$<0.0001, $^{***}P$<0.001, $^{*}P$<0.05.

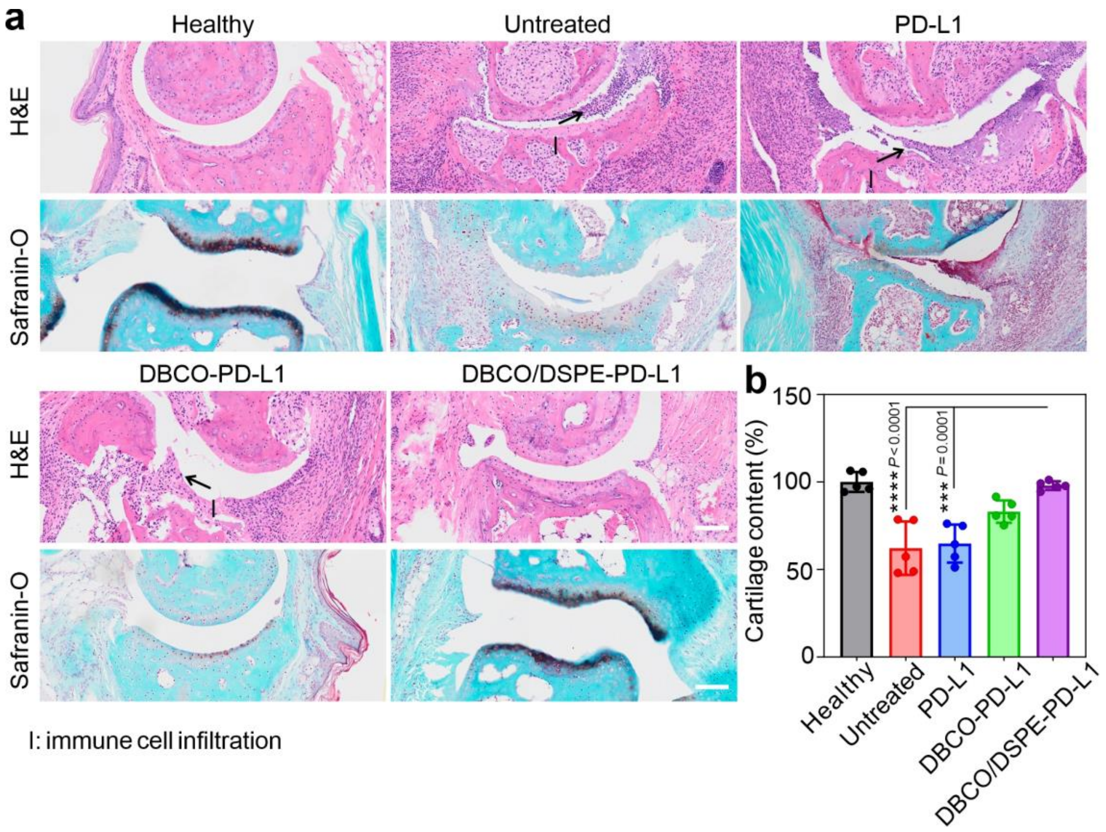

Supplementary Figure 38. ***In situ*** **immobilization of PD-L1 ameliorates digital joint disruption.** **a**, Representative images of H&E staining, safranin-O staining, and Masson staining on digital joints from mice with different treatments. **b**, Cartilage content quantified from safranin-O-stained sections of mice with different treatments. Scale bars: 100 μm. Data represent the mean ± s.d. (n = 5 biologically independent samples). The data were analyzed by one-way two-sided ANOVA; $^{****}P$<0.0001, $^{***}P$<0.001.

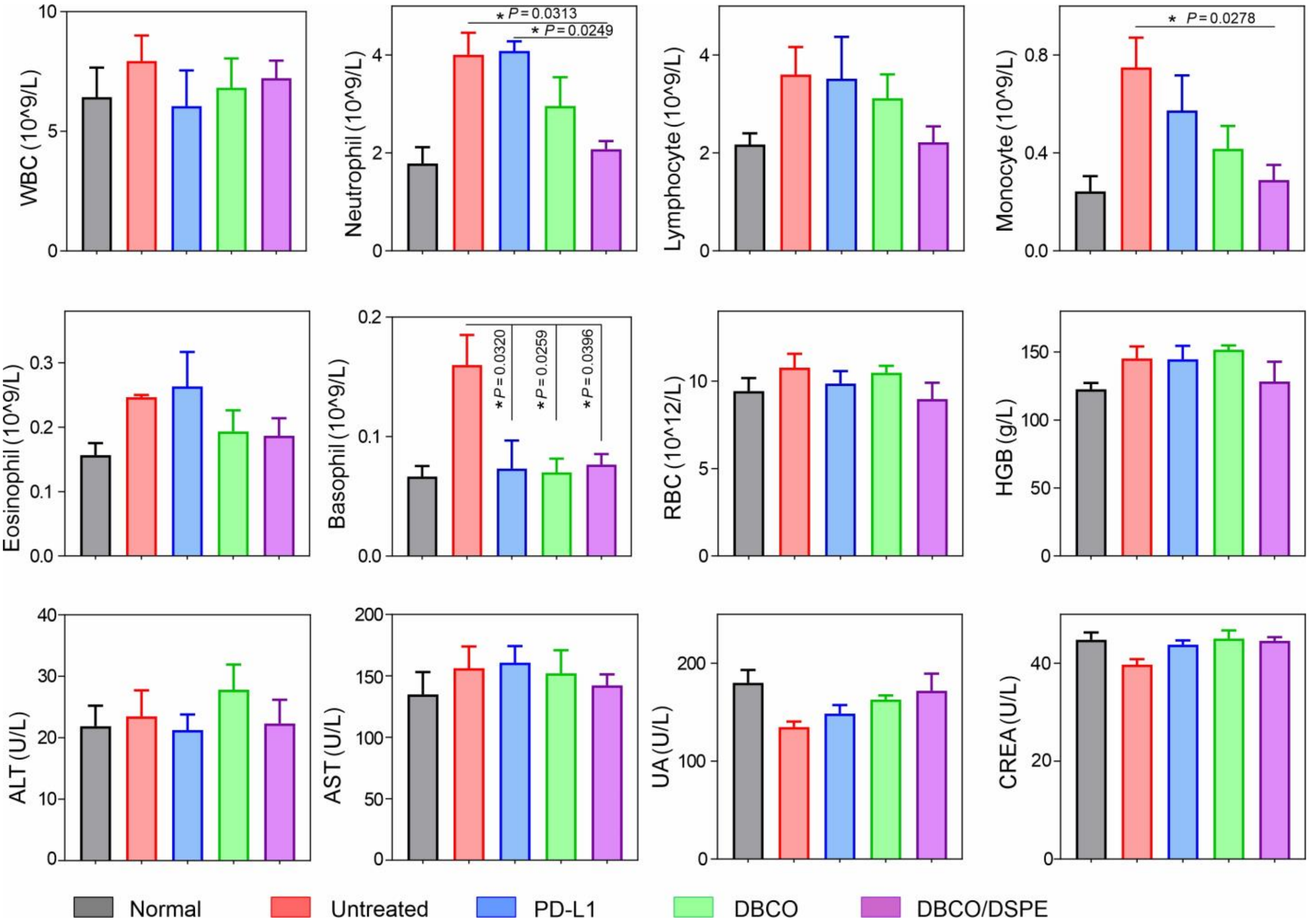

Supplementary Figure 39. Several key hematological, hepatic, and renal parameters of DBA mice with different treatments. Data represent the mean ± s.d. (*n* = 4 biologically independent samples). The data were analyzed by one-way two-sided ANOVA; $^{*}P<0.05$.

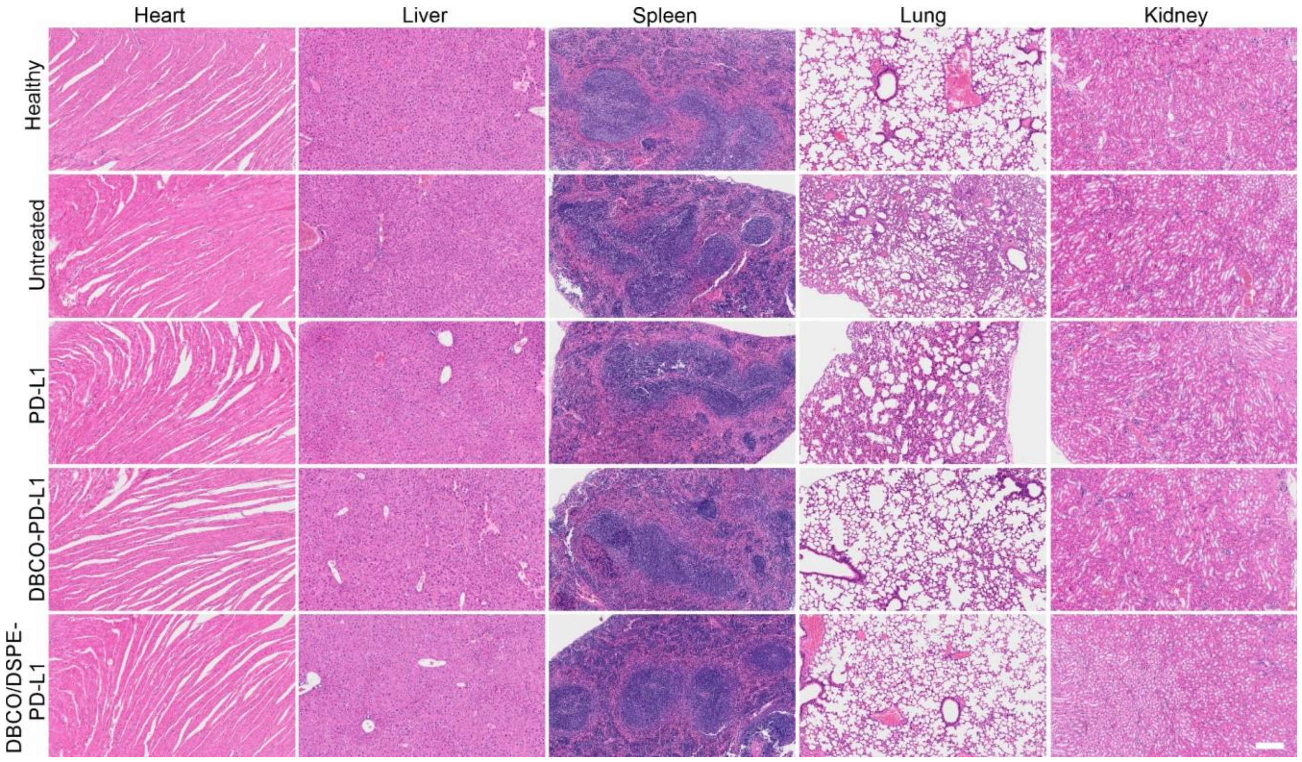

Supplementary Figure 40. ***In vivo* cytotoxicity evaluation.** H&E staining of hearts, livers, spleens, lungs, and kidneys harvested from DBA mice with different treatments. Scale bar: 200 µm.
